# Increase of niche filling with increase of host richness for plant-infecting mastreviruses

**DOI:** 10.1093/ve/veae107

**Published:** 2024-12-13

**Authors:** Sélim Ben Chéhida, Heemee Devi Bunwaree, Murielle Hoareau, Oumaima Moubset, Charlotte Julian, Laurence Blondin, Denis Filloux, Christophe Lavergne, Philippe Roumagnac, Arvind Varsani, Darren P Martin, Jean-Michel Lett, Pierre Lefeuvre

**Affiliations:** CIRAD, UMR PVBMT, St Pierre, La Réunion F-97410, France; CIRAD, UMR PVBMT, St Pierre, La Réunion F-97410, France; CIRAD, UMR PVBMT, St Pierre, La Réunion F-97410, France; CIRAD, UMR PHIM, Montpellier F-34090, France; PHIM Plant Health Institute, Université de Montpellier, CIRAD, INRAE, Institut Agro, IRD, Montpellier, France; CIRAD, UMR PHIM, Montpellier F-34090, France; PHIM Plant Health Institute, Université de Montpellier, CIRAD, INRAE, Institut Agro, IRD, Montpellier, France; CIRAD, UMR PHIM, Montpellier F-34090, France; PHIM Plant Health Institute, Université de Montpellier, CIRAD, INRAE, Institut Agro, IRD, Montpellier, France; CIRAD, UMR PHIM, Montpellier F-34090, France; PHIM Plant Health Institute, Université de Montpellier, CIRAD, INRAE, Institut Agro, IRD, Montpellier, France; Conservatoire Botanique National de Mascarin, St Leu, La Réunion F-97436, France; CIRAD, UMR PHIM, Montpellier F-34090, France; PHIM Plant Health Institute, Université de Montpellier, CIRAD, INRAE, Institut Agro, IRD, Montpellier, France; The Biodesign Center for Fundamental and Applied Microbiomics, Center for Evolution and Medicine, School of Life Sciences, Arizona State University, 1001 S. McAllister Ave, Tempe, AZ 85287-5001, USA; Structural Biology Research Unit, Department of Integrative Biomedical Sciences, University of Cape Town, Rondebosch, Cape Town 7700, South Africa; Division of Computational Biology, Department of Integrative Biomedical Sciences, Institute of Infectious Diseases and Molecular Medicine, University of Cape Town, Observatory 7925, South Africa; CIRAD, UMR PVBMT, St Pierre, La Réunion F-97410, France; CIRAD, UMR PVBMT, St Pierre, La Réunion F-97410, France

**Keywords:** ecology, viral metagenomics, *Mastrevirus*, host–pathogen interaction network, Poales, agroecosystem

## Abstract

Now that it has been realized that viruses are ubiquitous, questions have been raised on factors influencing their diversity and distribution. For phytoviruses, understanding the interplay between plant diversity and virus species richness and prevalence remains cardinal. As both the amplification and the dilution of viral species richness due to increasing host diversity have been theorized and observed, a deeper understanding of how plants and viruses interact in natural environments is needed to explore how host availability conditions viral diversity and distributions. From a unique dataset, this study explores interactions of *Mastrevirus* species (family *Geminiviridae*) with Poales order hosts across 10 sites from three contrasting ecosystems on La Réunion. Among 273 plant pools, representing 61 Poales species, 15 *Mastrevirus* species were characterized from 22 hosts. The analysis revealed a strong association of mastreviruses with hosts from agroecosystems, the rare presence of viruses in coastal grasslands, and the absence of mastreviruses in subalpine areas, areas dominated by native plants. This suggests that detected mastreviruses were introduced through anthropogenic activities, emphasizing the role of humans in shaping the global pathobiome. By reconstructing the realized host–virus infection network, besides revealing a pattern of increasing viral richness with increasing host richness, we observed increasing viral niche occupancies with increasing host species richness, implying that virus realized richness at any given site is conditioned on the global capacity of the plant populations to host diverse mastreviruses. Whether this tendency is driven by synergy between viruses or by an interplay between vector population and plant richness remains to be established.

## Introduction

The intuitive understanding that many viral crop pathogens have likely originated in, and emerged from, uncultivated “weeds” (Thresh 1978, Cooper and Jones 2006, Fargette et al. 2006, Elena et al. 2014, Roossinck and García-Arenal 2015) has prompted efforts to more thoroughly characterize the viruses that naturally associate with and infect uncultivated plant species (Muthukumar et al. 2009, Roossinck 2010, Bernardo et al. 2018). Over the past decade, multiple pioneering plant viral diversity studies in both natural environments and at agro-ecological interfaces between natural and cultivated areas have begun revealing the true scale of virus diversity associated with uncultivated plant species (Malmstrom and Alexander 2016, Bernardo et al. 2018, Susi et al. 2019, Valverde et al. 2020, Holmes 2022, McLeish et al. 2022). These ecosystem-scale studies have now firmly demonstrated that viruses are pervasive, and perhaps essential, components of global ecosystems (O’Malley 2016, Sullivan et al. 2017).

Owing to stark contrasts between the greater degree of macro-symptoms and the impact of viral infections observed in densely cultivated areas relative to those observed in diversified natural environments bordering these areas (García-Arenal and Zerbini 2019, Shates et al. 2019), questions have been raised about the relationships that exist between plant diversity, virus diversity, virus prevalence, and the manifestation of disease. While both infectious-disease risk increases (the amplification hypothesis) or decreases (the dilution hypothesis) with increasing host diversity (Keesing et al. 2006, Pagán et al. 2012, Faust et al. 2017, Liu et al. 2020, Gibson and Nguyen 2021) were reported, the relationship between virus diversity and host diversity remains elusive. Indeed, taking richness (i.e. counts of entities from the same taxonomic level) as a proxy of diversity, in some instances, higher viral richness was observed in plant species-rich areas than in poor areas (McLeish et al. 2022, Maclot et al. 2023), whereas in other situations, no clear correlation (Roossinck 2010) or a negative correlation was reported (Halliday et al. 2017, Susi and Laine 2021). These discordant observations can be potentially reconciled by acknowledging that viral species richness and community species (i.e. a group of species occupying the same geographical area at the same time) composition are likely influenced by multiple ecological factors that vary substantially from area to area and from season to season (Johnson et al. 2015, Martinez and Kline 2018, Valverde et al. 2020). Indeed, considering the complexity of the interactions occurring in host and viral communities, the interplay between viral and host species richness might be nontrivial. Accurate information on the viral–plant community structure detailing patterns of associations between viral and plant species would be required to properly interpret observed trends (Keesing et al. 2006). Despite the importance of precisely charting such plant–virus interaction networks to decipher such complex relationships, actual data on plant–virus associations from the field remain scarce (McLeish et al. 2019).

Recently, Claverie et al. (2023) focused on a single phytovirus genus (*Mastrevirus*, family *Geminiviridae*) to achieve such a detailed picture of plant–virus interactions in an agroecosystem (AE). Mastreviruses, all transmitted by leafhopper species in the genus *Cicadulina* (Hogenhout et al. 2008), have been known in La Réunion (a French overseas island in the Indian Ocean) since the 1970s (Baudin 1976). The first genomic sequence-confirmed report of *Mastrevirus* species on La Réunion was in 1986 with the description of maize streak virus (MSV, *Mastrevirus storeyi*)—the type species of the genus *Mastrevirus* (Peterschmitt et al. 1996)—on symptomatic maize plants. Subsequent studies have increased our knowledge of mastreviruses circulating on La Réunion, resulting in the description of host–pathogen interactions between eight virus species and 19 Poales species, with viruses displaying both diverse degrees of apparent host-specialization and frequent host-range overlaps (Claverie et al. 2023). Yet, it remains undetermined how environmental factors and plant composition might impact the host-range dynamics and species compositions of mastrevirus populations, as all previous observations were limited to AEs.

Here, to build on this remarkable knowledge of plant–virus effective host range in the field, we extended this survey to nine additional sites on La Réunion presenting with contrasted plant diversities. In doing so, we aimed to uncover *Mastrevirus* species diversity on La Réunion, provide insights into viral community structure, and investigate the ecological factors impacting the richness of viral populations, and the host ranges and spatial distributions of virus species. First, our observations suggest that the presence of mastreviruses on La Réunion is primarily due to the introduction of infected material from Africa. Second, there is a positive correlation between host richness and viral richness, uncovering a pattern of increase in niche occupancies with increased host richness. This original observation may stem from a synergy between viruses or the lifestyle of the insect vectors. Nonetheless, it also points to diverse environments, such as AEs, as ideal venues for plant viruses such as mastreviruses.

## Materials and methods

### Poales sampling

Poales samples were collected between November 2020 and April 2022 from 10 different sites across La Réunion (Supplementary Fig. S1, Table 1) spanning four AE sites [Bassin Plat (BP), Ligne Paradis (LP), Colimaçons (CL), and La Mare (LM)], three coastal grassland (CG) sites [Terre Rouge (TR), Pointe au Sel (PS), and Cap la Houssaye (CH)], and three subalpine grassland (SG) sites [Ligne d’Equerre (LE), Espace Naturel Sensible des Hauts de Mont-Vert (MV), and Piton de Caille (PC)]. AE sites are current experimental agricultural CIRAD stations with various cultivated species and frequent crop rotations. CG sites consist of floristically similar littoral meadows with a past grazing history. SG sites are former high-altitude pastoral meadows, now largely left unmanaged. Samplings were preferentially performed during rainy seasons. For each site, leaf samples were collected from up to 50 individual plants of each *Poales* species. In cases where fewer than 50 plants were found, samples were taken from all identified individuals. Following a random walk in the field, samples were collected regardless of potential infection symptoms (Supplementary Table S1), pooled by sites and species, dried overnight at 55°C, and stored at room temperature before use. The Conservatoire Botanique National Mascarin botanical database (https://mascarine.cbnm.org/) was used to classify plant species according to crop status (cultivated or uncultivated), life cycle (annual, which completes life cycle within one season; perennial, which lives more than a year; or annual/perennial for which life cycle depends on environmental conditions), and origin (native or introduced to La Réunion).

**Table 1.** Sampling sites and summary of sampled pools for each campaign.

Type of Site	Site	Lat.	Lon.	Alt. (m)	November 2020	January 2021	April 2021	November 2021	April 2022	Total
CG	TR	−21.3498	55.4975	31	0	0	4 (1)	6	7	17 (1)
CG	PS	−21.2024	55.2814	8	0	0	9	6 (1)	11 (2)	26 (3)
CG	CH	−21.0187	55.2352	15	0	0	13 (1)	5	17 (2)	35 (3)
AE	LM	−20.9032	55.5311	70	0	0	0	0	18 (8)	18 (8)
AE	BP	−21.3216	55.4892	166	4	13 (1)	15 (9)	16 (2)	22 (10)	70 (22)
AE	LP	−21.3166	55.4873	180	0	0	0	10 (2)	17 (4)	27 (6)
AE	CL	−21.1304	55.3054	819	0	0	13 (1)	9 (3)	15 (1)	37 (5)
SG	LE	−21.2916	55.5733	1062	5	0	0	8	0	13
SG	MV	−21.2859	55.6030	1586	7	0	0	6	0	13
SG	PC	−21.1902	55.6380	2130	9	0	0	8	0	17
**Total**					25	13 (1)	54 (12)	74 (8)	107 (27)	273 (48)

For each sampling date, the number of collected plant species pools are indicated. The number in parentheses refers to the number of pools for which complete Mastrevirus genomes were obtained.

### Insect sampling

To obtain an overview of the diversity of insects in the order Hemiptera, including vectors of mastreviruses, five bulk collections of insects were conducted at the TR, BP, LE, MV, and PC sites, simultaneously with plant collection. For each site, insects were collected using a vacuum device at five separate points containing *Poaceae* species, with sampling conducted for five minutes along the entire height of the grass cover.

### Total DNA extraction, MinION sequencing, and read assemblies

After sample homogenization using the TissueLyser II (Qiagen, Hilden, Germany), total DNA was extracted from 20 mg of plant material using the DNeasy Plant Pro DNA extraction kit (Qiagen, Hilden, Germany), following the manufacturer’s instructions. DNA extracts were stored at −20°C before use. The rolling circle amplification (RCA)-MinION protocol (Ben Chehida et al. 2021) was then used, employing the *EquiPhi29* DNA polymerase (Stepanauskas et al. 2017) kit (ThermoFisher Scientific, USA) for RCA. For each sequencing run, 10 samples were multiplexed along with a positive control (DNA extract of a *Solanum lycopersicum* plant infected with tomato yellow leaf curl virus—*Begomovirus coheni*, family *Geminiviridae*) and a negative control (DNA extracted from a healthy uninfected tomato plant) on a single Flongle (FLO-FLG001). High-accuracy basecalling and demultiplexing were performed using Guppy v4.2.2 (https://nanoporetech.com/). Sequences of members of the *Mastrevirus* genus (in the phylum *Cressdnaviricota*), *Alphasatellitidae*, and *Tolecusatellitidae* (two families of satellite molecules associated with members of the phylum *Cressdnaviricota*) genomic components were assembled using two complementary pipelines. The first assembly pipeline is described by Ben Chéhida et al. (2021). In the second assembly pipeline, high-quality reads were used as queries to search a cressdnavirus and satellite molecule reference sequence database (obtained from GenBank in January 2023) using BLASTn. Filtered reads were used for assembly using Flye 2.6 (Kolmogorov et al. 2019), Wtdbg 2 2.5 (Ruan and Li 2020), and Miniasm v0.3 (https://github.com/lh3/miniasm). After first “polishing” using Flye, contigs were circularized using custom scripts when appropriate. Sequences obtained from the three assembly programs were clustered and merged into consensus sequences before a second step of polishing using Flye. Final polishing of the contigs was performed using Medaka v1.2.2 (https://github.com/nanoporetech/medaka). All contigs obtained from both approaches were subjected to a BLASTn search against the National Center for Biotechnology Information nt database for preliminary species assignment. Coverage statistics were obtained using minimap2 v2.17 (Li and Birol 2018). Only contigs representing a minimum of 2% of the total raw reads per sample were considered for further analysis.

### Full genome cloning and Sanger sequencing

Samples presenting with sequences from putative new viral species (following the International Committee on Taxonomy of Viruses species demarcation guidelines, https://ictv.global/report/chapter/geminiviridae) or from viral species newly identified on La Réunion were submitted to RCA-RFLP for full genome cloning and Sanger sequencing as described in Inoue-Nagata et al. (2004). Here, 1 µl of the RCA product was digested using ApaLI, KpnI, NcoI, SalI, or XbaI to yield a ∼2.7-kb fragment, which was set up for Sanger sequencing as described in Ben Chéhida et al. (2021). Full-length mastrevirus genomes were then assembled using Geneious Prime 2022.2 (http://www.geneious.com).

### Phylogenetic and recombination analyses

All the *Mastrevirus* species reference full genome sequences (as obtained from GenBank RefSeq in January 2023; Supplementary Table S2) along with all the sequences obtained from samples collected on La Réunion and those identified in this study were aligned using MAFFT v.7 (Katoh and Standley 2013). A maximum likelihood phylogenetic tree was inferred using FastTree v2.1.18 (Price et al. 2010) with the generalized time-reversible model and Shimodaira-Hasegawa test for branch support. The tree was edited using the APE R package (Paradis et al. 2019) with *Eragrostis curvula* streak virus (NC_012664.1) used as the out-group. Additional trees for encoded capsid protein (CP) and replication-associated protein (Rep) amino acid sequences were similarly obtained.

### Viral community diversity and structure analyses

Site differences in plant sample species richness were calculated with the Jaccard distance on binary data using the *vegdist* function of the “vegan” R package (Oksanen et al. 2019). The ordination of the sites by observed Poaceae species was projected in two dimensions with principal coordinates analysis using the cmdscale function (“vegan” R package). The optimal number of clusters was calculated using the *fviz_nbclust* function from the “factoextra” R package (Kassambara and Mundt 2020) and clustering at a 95% confidence level was performed using the *kmeans* function (“stats” R package).

Plant characteristics (crop status, life cycle, and origin) across all sites were compared between sites using Fisher exact tests. Pairwise comparisons for proportions (“stats” R package) based on the Benjamini and Hochberg (1995) method were calculated when globally significant differences were found. Differences in viral richness between the different site types according to the host–plant characteristics were assessed using the Kruskal–Wallis rank sum test (Hollander and Wolfe 1973) (“stats” R package). Three tests were performed with a 95% confidence level.

From the binary plant–virus interaction matrix, a bipartite network was generated using the R “bipartite” package (Dormann et al. 2008). Three measures were used to further characterize the virus–host community (Newman 2003, Moury et al. 2021). The assortativity (the tendency for nodes to connect to other nodes with similar properties), among the plant tribes, origin, and plant life cycle, was obtained using the *assortativity_nominal* function of the “igraph” R package (Csardi and Nepusz 2006); the nestedness (the tendency for nodes with fewer connections to interact with subsets of the partners of nodes with more connections) was computed using the “NODF2” method (Almeida-Neto et al. 2008) of the *nested* function (“bipartite” R package); and the modularity (the measure of the strength of division of a network into subgroups of interacting nodes) was calculated using the *computeModules* function with the “Newman” method (Newman 2004, Beckett 2016) (“bipartite” R package). These algorithms proved to be suitable for binary network analysis (Newman 2003, Almeida-Neto et al. 2008). Their statistical significances were estimated after comparison with 1000 random permutations of the matrix using “quasiswap” null models (“none,” “rows,” “columns,” and “both”; “vegan” R package). The “none” model preserves the total number of interactions, the “rows” model preserves the sum of interactions per plant species, the “columns” model preserves the sum of interactions per virus species, and the “both” model preserves the sum of interactions per plant and virus species.

## Results

### Extending our knowledge of mastreviruses diversity

A sequencing protocol specifically devised for small circular DNA viruses was applied to all the pools of plants collected during the surveys. While most of the genomic sequences obtained were those of mastreviruses (65/90), 22 contigs were assigned to the family *Genomoviridae* and 3 to *Alphasatellitidae* (Sorghum mastrevirus–associated alphasatellite—*Somasatellite sorghi*) (Supplementary Table S3). A total of 65 complete genome assemblies from 12 different mastreviruses were obtained, with 8 having been previously characterized, 3 being putative new members of the genus *Mastrevirus* (Supplementary Figs S2 and S3; Supplementary Table S4), and 1 presenting with a defective/subgenomic sequence (i.e. the sequence present with open reading frames (ORFs) encoding a movement and CPs, but no ORF encoding a Rep) of a probable new species (Supplementary Tables S4 and S5). Urochloa streak virus (*Mastrevirus urochloae*), which was found infecting six Poaceae species in this study, is reported for the first time from La Réunion (Fig. 2; Supplementary Table S5). The three putative new *Mastrevirus* species were tentatively named *Cenchrus echinatus*–associated virus (CEAV; tentative species name *Mastrevirus cenchri*), *Cenchrus purpureus* mild streak virus (CPMSV; tentative species name *Mastrevirus purpurei*), and *Urochloa decumbens*–associated virus (UDAV; tentative species name *Mastrevirus urochloareunionense*) (Supplementary Fig. S2; Supplementary Tables S4 and S5). The three viruses presented with typical mastrevirus genome organizations with nonanucleotide characteristics of geminivirus virion strand origins of replication (V-ori) and ORFs likely encoding movement protein, CP, and Rep (Supplementary Table S4). The putative subgenomic mastrevirus sequence (DefMS) was obtained from a *Saccharum spp*. pool (Supplementary Table S5). It possessed a mastrevirus-like V-ori nonanucleotide (TAATATTAC) and two ORFs likely encoding a movement protein and CP. No inter-specific recombination event was detected in the newly characterized species.

### Environment type impacts proportions of mastreviruses-infected Poales species

**Figure 1.**
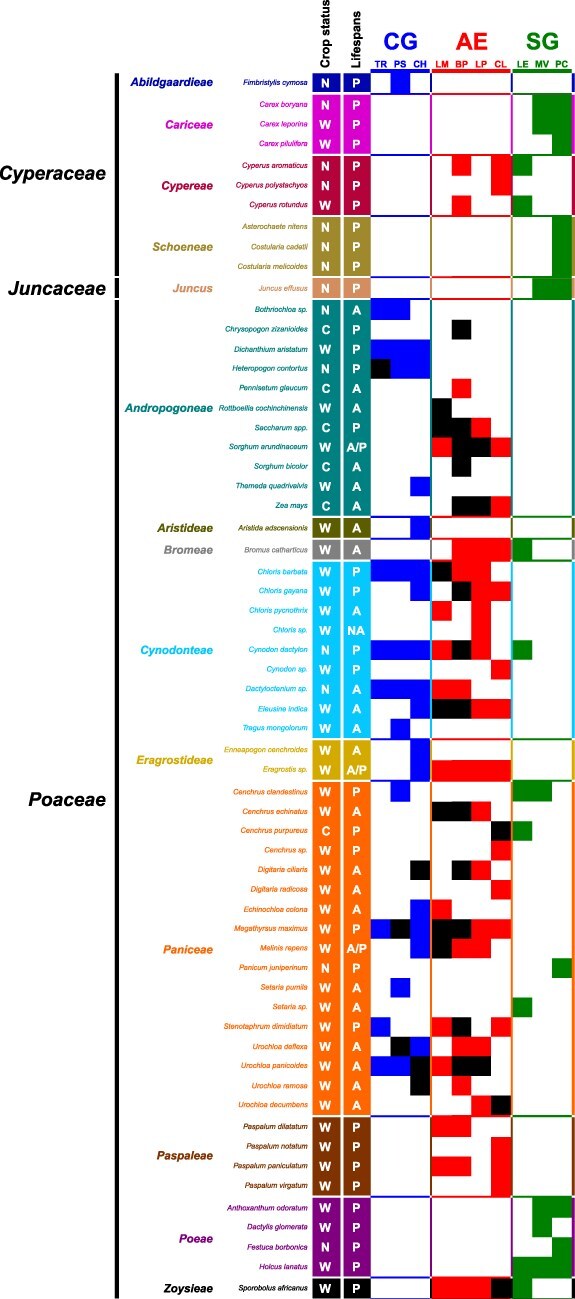
Presence/absence matrix of the *Poales* species (rows) in the different sites (columns). The *Poales* species are organized by families (*Cyperaceae, Juncaceae*, and *Poaceae*) and colored and organized according to the classification of tribes. Origin and crop status (N: uncultivated native; W: uncultivated introduced; C: cultivated introduced) and life cycles (A: annual; P: perennial; A/P: annual or perennial; NA: unknown) are indicated in the first and second columns, respectively. Light colored squares indicate the presence of the *Poales* species. Black squares indicate the detection of mastreviruses for the corresponding species.

Of all the 273 pools of individual plant species (collectively representing >13 000 individual plants; Table 1; Supplementary Table S1), cressdnaviruses and/or known satellites of cressdnaviruses were found in 48 (together representing 22 of the 61 collected plant species; Supplementary Tables S3 and S6). None of the 10 Cyperaceae and the single Juncaceae species (together representing 24 plant sample pools) contained detectable evidence of cressdnaviruses. All the positive plants belonged to the Poaceae family (Fig. 1).

In total, 2/14 (14%) uncultivated native species, 15/41 (37%) uncultivated introduced species, and 5/6 (83%) cultivated introduced species were hosts of mastreviruses (Fig. 1). While most of the hosts were uncultivated introduced species, there were no significant differences between the viral species richness obtained from the cultivated and uncultivated species (Kruskal–Wallis test: *P*-value = 1.9 × 10^−1^) or from the native and introduced species (Kruskal–Wallis test: *P*-value = 1). When comparing annual versus perennial Poaceae species, 10/22 (45%) of the annual, 10/33 (30%) of the perennial, and 2/3 (67%) of the annual/perennial species were host for mastreviruses, but no significant difference was observed between the groups either in the proportion of plant species (Fisher’s exact test: *P*-value = 2.6 × 10^−1^) harboring mastreviruses or in the degree of viral species richness that they harbored (Kruskal–Wallis test: *P*-value = 9.1 × 10^−1^).

Regarding the sites, it must be noticed first that strong differentiation in plant composition exists from one type of site to the other (Supplementary Fig. S1C). Globally, sites shared a mean of 51% of the species with sites of the same types, while they shared a mean of 18% of the species with sites of other types. Plant characteristics from site to site were also contrasted. In accordance with expected decreases in numbers of introduced species with increasing altitude (Fenouillas et al. 2021), there was a significant difference in the proportions of native species between the different site types (Supplementary Tables S1 and S2; Fisher’s exact test: *P*-value = 1.0 × 10^−2^). Additionally, a marginally significant difference was observed with respect to the proportions of annual, perennial, and annual/perennial plant species observed at the sites (Supplementary Tables S1 and S2; Fisher’s exact test: *P*-value = 5.6 × 10^−2^).

While 34/56 (61%) of the Poales species at AEs and 15/24 (63%) at CGs were mastrevirus hosts, 32/34 (94%) of the pools of hosts (i.e. plants found in our study or previous studies in La Réunion as infected with mastreviruses) were detectably infected with mastreviruses at AEs and 6/15 (40%) at CGs (Fig. 1). It is noteworthy that no mastreviruses were found in any plant pools collected from SGs, despite the presence of leafhoppers (Cicadellidae) at all SGs and the sampling of abundant *Cicadulina mbila* at the LE site (Supplementary Table S7).

### The minimum host–virus interaction network

**Figure 2.**
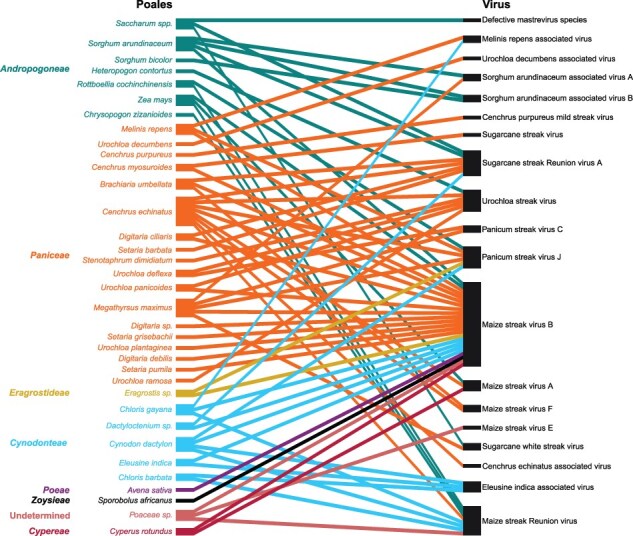
Bipartite interaction graph representing the association between *Poales* species (left side of the diagram) and viral species and strains (right side of the diagram) build from all the available data from this work and previous studies. *Poales* species are colored and organized by tribe.

Building on the previous data obtained from La Réunion [all from AEs, including data obtained in Claverie et al. (2023)] and the original data obtained here from a wider variety of sites, we attempted to construct the most complete network of virus–host species interactions (Fig. 2). The virus–host species interaction network represents 74 interactions between 35 plant species belonging to seven tribes and 14 viral species (or 19 viral entities when considering strains). Individual plant species were found hosting between one (for 14 plant species) and eight virus species (for *Cenchrus echinatus*) with a mean number of two virus species per host (Supplementary Table S5). Among all the mastreviruses, five have only ever been sampled on La Réunion from a single host species. Thirteen other virus entities were found infecting two or more different host species with MSV-B infecting the highest number of different host species (23/35).

In order to determine whether the interactions represented by the network bear imprints of the mastreviruses ecology, three different structural features of the virus–host species interaction network—assortativity, nestedness, and modularity—were tested for their deviations to expectations under models of random virus–host species interactions. Assortativity analysis (Table 2) revealed no association between the network structure and the classification of hosts according to whether they were native or introduced species (*P*-values of .314 and .511) or whether they were annuals, perennials, or annual/perennials (*P*-values of .189 and .471).

**Table 2.** Analysis of assortativity of Poales–Mastrevirus interaction matrices.

		Null model			
Plant characteristic	Assortativity score	None	Rows	Columns	Both
Tribe	−1.4 × 10^−3^	.864	.806	**.990**	**.992**
Origin^a^	−4.6 × 10^−2^	.511	.473	.332	.314
Life cycle^b^	−5.1 × 10^−2^	.471	.423	.202	.189

The probability value (*P*-value) indicates the frequency of the actual matrix showing a strictly higher assortativity score than that of null-model matrices. *P*-values > 0.95 (significant assortativity) are in bold.

a Native or introduced.

b Annual, perennial, or annual/perennial.

However, the tribes to which host species belonged contributed substantially to defining the structure of the network with higher assortativity (meaning a higher tendency for plants from the same tribe to host similar virus entities) obtained in comparison to most null models employed for permutation (*P*-value ≥ .990; Table 2). While this implies specialization and suggests the existence of some modularity within the network, no significant degrees of modularity were observed (Table 3). Instead, significant degrees of nestedness were detected within the network (*P*-value ≥ .981; Table 3), indicating that the host ranges of some viruses tended to be embedded within the host ranges of other viruses.

**Table 3.** Analysis of nestedness and modularity of Poales–Mastrevirus interaction matrices with, respectively, NODF2 and Newman algorithm.

	NODF2					Newman algorithm				
		Null model					Null model			
Matrix	Nestedness score	None	Rows	Columns	Both	Modularity score	None	Rows	Columns	Both
CG	38.89	.644	.644	.44	.502	.44	.930	.752	.830	.287
AE	34.58	**1**	**1**	**.981**	.460	.45	*.005*	*0*	.283	*.011*
Reunion	30.52	**1**	**1**	**.991**	.430	.48	.061	*0*	.737	.256

The probability value (*P*-value) indicates the frequency of the actual matrix showing a strictly higher nestedness or modularity score than that of null-model matrices. *P*-values > 0.95 (significant nestedness or modularity) are in bold and *P*-values ≤ 0.05 (significant anti-nestedness or anti-modularity) are in italic.

### Not all interactions are achieved in the realized network

Globally, 24, 15, and 4 mastrevirus hosts were found at the AEs, CGs, and SGs, respectively (Fig. 1; Supplementary Table S5). Apart from the MV and PC sites (both SGs), where no hosts were sampled, the other eight sampling sites ranged from 3 to 18 mastrevirus hosts (Fig. 1; Supplementary Fig. S3). The analysis of virus richness among sites revealed that there was a positive correlation (Pearson’s *R*^2^ = 0.81; *P*-value = 4.2 × 10^−4^) between the number of host species present and the number of mastreviruses detected at the sampling sites (Supplementary Fig. S4).

**Figure 3.**
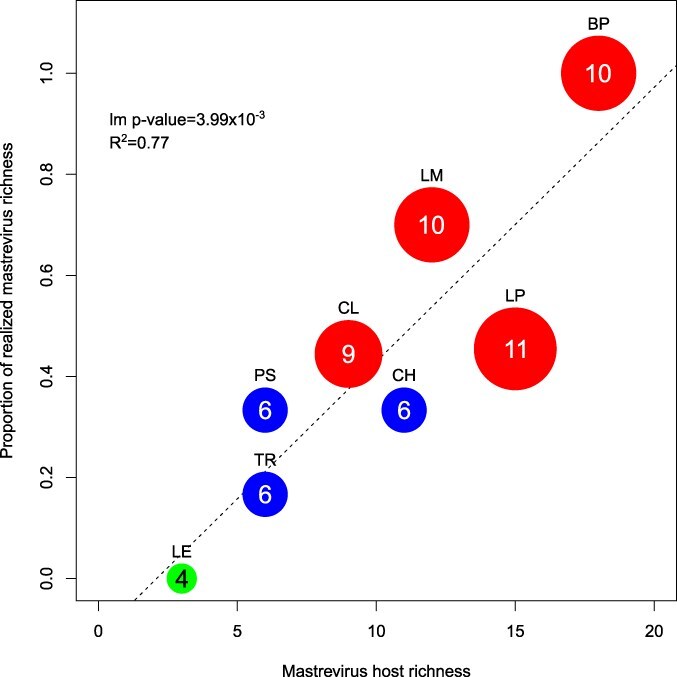
Proportion of realized *Mastrevirus* species richness as a function of plant host richness. Sites are presented as colored dots (red for AE sites, blue for CG sites, and green for SG sites) whose sizes are proportional to the maximum number of *Mastrevirus* species that could have been observed knowing the plant virus diversity of the site. This maximum number of species is also indicated on each dot. For each site, the proportion is obtained by dividing the realized number of *Mastrevirus* species with the maximum number of *Mastrevirus* species achievable. Linear regression is indicated with the dotted line. The *P*-value for the linear regression slope to be different from zero is indicated on the top left.

Going one step further, we calculated the maximum number of viral species that could have been detected at each sampling site—which are contingent on the host species that were present at each site—and calculated the proportions of these viruses that were observed at each of the sites. These proportions, i.e. the percentages of realized viral richness, were ranging from 0% for the LE site, with none of the four mastreviruses that could have infected the three hosts present in that site being detected, to 100% for the BP site, with all the 10 potential mastreviruses being detected. It must be noted here that no mastreviruses were expected from two of SGs (MV and PC). Globally, the percentages of realized viral richness was higher for AEs (62%) than for CGs (27%). Importantly, using per-site results, there was a significant correlation (Pearson’s *R*^2^ = 0.77; *P*-value = 3.99 × 10^−3^) between the realized *Mastrevirus* species richness and the richness of mastrevirus host species found at those sites (Fig. 3; Supplementary Fig. S4).

We later thought to analyze the numbers of virus–host species interactions that were observed at each of the sampling sites as a function of the fraction of the maximum number of interactions that could have been observed at the sites (Supplementary Fig. S4). In doing so, we accounted for the fact that some of the mastreviruses can infect multiple host species and thus counted the number of distinct virus–host infections rather than the number of viral species detected. These “realized interaction proportions” varied from 0% for the LE site to 100% for the CL site. At the BP site (where the highest number of *Mastrevirus* species was observed), 67% of all the virus–host species interactions that could have been observed at the site were observed. While the numbers of interactions were significantly correlated with *Mastrevirus* species richness (Pearson’s *R*^2^ = 0.94; *P*-value < 1.00 × 10^−5^; Supplementary Fig. S4) and *Mastrevirus* host species richness (Pearson’s *R*^2^ = 0.65; *P*-value = 4.94 × 10^−3^; Supplementary Fig. S4), there was no detectable correlation between the realized interaction proportions and either richness of observed *Mastrevirus* species (Pearson’s *R*^2^ = 0.18; *P*-value = 3.49 × 10^−1^, Supplementary Fig. S4) or richness of mastrevirus host species (Pearson’s *R*^2^ = 0.01; *P*-value = 8.53 × 10^−1^; Supplementary Fig. S4). This suggests that the number of host species infected by a given virus at a site was not obviously influenced by either the numbers of host species or the numbers of different *Mastrevirus* species found at that site.

## Discussion

### Viral diversity patterns provide insight on the history of mastreviruses in La Réunion

From the analysis of the sampled pools, all the *Mastrevirus* species, except the *Melinis repens*–associated virus (*Mastrevirus melinis*) and sugarcane streak virus (*Mastrevirus saccharofficinari*), were identified. It must be noticed that, in accordance to Claverie et al. (2023) findings, no MSV-A were identified. While additional mastreviruses were characterized from our surveys and would in turn increase our knowledge of the global African streak virus (AfSV) diversity, it is important to notice that no *Mastrevirus* species was detected from SGs. SG sites were at higher altitude, implying that seasonal temperature variations might result in pauses in transmission cycles (van Rensburg et al. 1991, Reynaud et al. 2009). However, the presence of abundant *C. mbila*, one of the most important vector species (Webb 1987), at the LE site and the presence of leafhoppers in SGs suggest that AfSV transmission by these insects is possible for these sites. Indeed, MSV transmission has been proven at altitudes up to 1300 m on La Réunion (Reynaud 1988). Combining this observation with the higher proportion of native Poales at SGs (8/20) compared to that of other types of sites, it would indicate that there is no AfSV that adapted and coevolved with the native (frequently endemic) Poales found at high-altitude sites. Conversely and consistent with previous findings (Claverie et al. 2023), AfSV species found on La Réunion were largely associated with uncultivated introduced plant species. This, together with the observation that 6/13 of the *Mastrevirus* species that have so far been detected on La Réunion are also found on mainland Africa, is largely consistent with the hypothesis that *Mastrevirus* species were introduced to the island together with their alien hosts from Africa after the first human establishment on the island in the 17th century (Kopp 1930, Monjane et al. 2011). The most probable step-wise additions of hosts along with their viral corteges would in turn result in the making of the global virus–host network we have uncovered.

### Virus–host interactions bear imprints of community wide process

As the network we constructed was assembled from data accumulated by multiple studies, neither the timings of collections nor the precise sampling sites were taken into account. We would therefore consider this network to represent the overall minimal host ranges of the *Mastrevirus* species found on La Réunion, a network that would expand if more surveys on more sites were conducted. Indeed, we understood that (i) it is very likely that our sampling has been too sparse to detect all of the actual interactions; (ii) we may have failed to identify all the host plants either because of low infection frequencies or plant misidentification; (iii) it is very unlikely that, even with exhaustive sampling and perfect detection methods, examples of all possible interactions would be present at a given time at a given site; and (iv) the actual structure of the network might not be static, but instead change over time as new viral and host lineages emerge, spread, and intermingle across the sampling sites (Valverde et al. 2020).

Within the network, 6 of the virus entities were found to infect a single host, viruses, and could be referred to as specialists, and 13 others were found infecting two or more hosts belonging to two or more different plant tribes (or genera) and could be considered as generalists. However, some of the plant species are very closely related (i.e. the plant species are not all equally different from one another), and as such four additional virus entities could therefore be considered as specialists despite these virus entities having been isolated from different species (Supplementary Table S5). As different host ranges were observed, we analyzed the patterns of assembly of hosts and viruses to inform on the community functioning. Nestedness was significant when compared to most permutations sets but the most stringent (the “both” null model, that here, hardly generates deviation from the original data due to the small size of our network). Nestedness is frequently observed in host–parasite communities (Flores et al. 2011, Beckett 2016). It has been suggested that nestedness would be detected in robust (Memmott et al. 2004, Bascompte et al. 2006) and species-rich networks (Bastolla et al. 2009). Conversely, no modularity was detected (even some instances of anti-modularity were found, i.e. less modularity than expected). If significant degrees of modularity had been detected within the network, it would have been a signal of strong host–virus associations that could lead eventually to less robust and more species-poor networks (Grilli et al. 2016, Dormann et al. 2017, Valverde et al. 2020). It has been hypothesized that community composition stability is a prerequisite for the emergence of modularity (Beckett 2016, Valverde et al. 2020). Here, the plant community is composed of 57% (20/35) annual species (6/35 cultivated and 14/35 uncultivated; Fig. 1; Supplementary Tables S5 and S6). These annual species account for 62% (46/74) of the interactions within the network. While the cultivated annual species are periodically harvested and removed from the ecosystem, the annual uncultivated species naturally die-off with natural seasonal cycles. It is plausible that this removal may prevent the emergence of strong modular interactions within the interaction network. The 20 annual plant species considered here include the unique hosts of four of the “specialist” mastreviruses (CEAV, CPMSV, UDAV, and DefMS): all of which have been described here for the first time. CPMSV and DefMS are associated with vegetatively propagated plants (*Cenchrus purpureus* and sugarcane, respectively), which may preclude the requirement for an insect vector and/or alternative hosts for these species. However, the hosts of CEAV and UDAV (*C. echinatus* and *U. decumbens*, respectively) are not vegetatively propagated, which suggests that, in the absence of seed transmission, these viruses might require either currently unidentified alternative host species or a long-lived vector species within which they can overwinter. The 10 other *Mastrevirus* species that are represented within the network were each found infecting two or more different host species, at least one of which was an uncultivated perennial host that could, conceivably, facilitate the persistence of the viruses across the seasons.

### Rich gets richer relationship between mastreviruses and host richness

Bearing in mind that the virus species accounted for in our network display distinct (although in some cases overlapping) host ranges, it was expected that more host species at a site would be conducive to the discovery of more *Mastrevirus* species at that site (Kamiya et al. 2014). Consistent with this anticipation and similar to our findings, it has been observed that, irrespective of the particular viral genus being considered, the numbers of detectable Poaceae-infecting virus species increase with increasing numbers of sampled Poaceae species (Susi and Laine 2021). Conversely, the positive correlation between the realized *Mastrevirus* species richness and the richness of mastrevirus host species, was less anticipated. Indeed, it indicates a rich gets richer relationship, entirely compatible with an amplification effect, where higher niche fillings are observed in sites presenting with higher host richness than sites presenting with lower host richness (Supplementary Fig. S4). Increasing host diversity and potentially “optimal” host richness would in turn potentially foster the maintenance of higher viral richness. While this could indicate that synergism between virus species might occur, the absence of an obvious correlation between the observed numbers of virus–host interactions and the observed host or viral richness (i.e. the realized interaction proportions) may be indicative of a limited effect, if any, of the phenomenon.

Finally, it is entirely possible that increases in observed viral richness are more directly associated with environmental conditions that are more favorable for virus transmission (i.e. conditions that foster the proliferation and survival of vector species): both dilution and amplification effects have been theorized and observed for vector-borne pathogens (Rohr et al. 2020). The relationship between host and virus species richness is nontrivial and is dependent on vector feeding behavior and the specifics of virus transmission (Keesing et al. 2006, Miller et al. 2013, Faust et al. 2017). For mastreviruses, the absence of definitive information on the vectors, especially on La Réunion, limits our ability to interpret the observed pattern.

## Concluding remarks

Here, we find that the distribution of Poaceae-infecting mastreviruses found in different ecological contexts on La Réunion is consistent with the viruses having originated from multiple, likely human-mediated, introductions to the island of African Poaceae species and their associated viral species. While we lack the suitable sequence sets for most of the identified *Mastrevirus* species (Monjane et al. 2011), further model-based phylogeographic studies would be required to refine that hypothesis.

The patterns of mastrevirus and host species richness and the interactions that are observed to occur indicate that the mastreviruses, their plant host species, and their insect vector species form a community that is likely most suitable for the survival and propagation of viruses within agro-ecological contexts. For example, low altitude environments with large numbers of introduced Poaceae species that are capable of hosting mastreviruses and grow in close proximity to native Poaceae species that are also capable of hosting mastreviruses.

Our analyses of virus–host interaction patterns and their associations with *Mastrevirus* species richness suggest that it may be necessary to obtain a better understanding of the impacts of environmental factors on viral transmission dynamics to more fully explain why virus–host interactions that are known to be possible are frequently undetectable at field sites that are otherwise apparently conducive to the occurrence of these interactions. However, the discovery of an increase in niche filling with an increase in host richness might be at the root of this observed difference between the possible and realized host–virus interactions. While it may be needed to obtain quantitative data on infection rates, host qualities, circulating vectors, and viruliferous status of vectors to more finely understand this relationship, it opens a research avenue to understand how certain arthropod-transmitted viruses, such as most of the phytoviruses, thrive in certain environments, such as those of the AEs.

## Supplementary Material

veae107_Supp

## Data Availability

Sequences described in this study are available on GenBank under the accession numbers PQ434707–PQ434771 for the full genome sequences obtained after MinION read assembly, and OQ451138–OQ451142 for the full genome sequences obtained after Sanger sequencing assembly. Publicly available datasets were analyzed in this study.
