# Correction to: Increase of niche filling with increase of host richness for plant-infecting mastreviruses

**DOI:** 10.1093/ve/veaf046

**Published:** 2025-06-16

This is a correction to: Sélim Ben Chéhida, Heemee Devi Bunwaree, Murielle Hoareau, Oumaima Moubset, Charlotte Julian, Laurence Blondin, Denis Filloux, Christophe Lavergne, Philippe Roumagnac, Arvind Varsani, Darren P Martin, Jean-Michel Lett, Pierre Lefeuvre, Increase of niche filling with increase of host richness for plant-infecting mastreviruses, *Virus Evolution*, Volume 10, Issue 1, 2024, https://doi.org/10.1093/ve/veae107

In the version originally published, the correspondence address for Pierre Lefeuvre was incorrectly listed as: ``Institut de Biologie Moléculaire des Plantes, CNRS UPR2357, Université de Strasbourg, 67 084 Strasbourg, France.'' This has been corrected in the article to read: ``CIRAD, UMR PVBMT, St Pierre, La Réunion F-97410, France.''

