# Supplementary material for: Increase of niche filling with increase of host richness for plant-infecting mastreviruses
Source: Virus Evol. 2024 Dec 13;10(1):veae107. doi: 10.1093/ve/veae107 (PMC11665825; doi:10.1093/ve/veae107)
Supplement: veae107_Supp [file veae107_supp.zip › Supp/Supplementary Table .docx]

**Supplementary Table 1. Summary of sampled pools in each type site and site for each sampling date.** For each site, leaf samples were collected from up to 50 individual plants of each Poales species. In cases where fewer than 50 plants were found, samples were taken from all identified individuals.

| **Date Sampling** | **Type of Site** | **Site** | **Poales Species** | **Pool Id** | **N Samples** |
| --- | --- | --- | --- | --- | --- |
| Nov 2020 | AE | BP | *Chloris gayana* | 20_REU_E0102 | 50 |
| Nov 2020 | AE | BP | *Cyperus rotundus* | 20_REU_E0103 | 50 |
| Nov 2020 | AE | BP | *Cynodon dactylon* | 20_REU_E0109 | 50 |
| Nov 2020 | AE | BP | *Bromus catharticus* | 20_REU_E0111 | 50 |
| Nov 2020 | SG | LE | *Bromus catharticus* | 20_REU_E0303 | 50 |
| Nov 2020 | SG | LE | *Cyperus rotundus* | 20_REU_E0304 | 50 |
| Nov 2020 | SG | LE | *Holcus lanatus* | 20_REU_E0306 | 50 |
| Nov 2020 | SG | LE | *Cynodon dactylon* | 20_REU_E0311 | 50 |
| Nov 2020 | SG | LE | *Cenchrus clandestinus* | 20_REU_E0312 | 50 |
| Nov 2020 | SG | MV | *Dactylis glomerata* | 20_REU_E0401 | 50 |
| Nov 2020 | SG | MV | *Holcus lanatus* | 20_REU_E0406 | 50 |
| Nov 2020 | SG | MV | *Carex leporina* | 20_REU_E0408 | 50 |
| Nov 2020 | SG | MV | *Juncus effusus* | 20_REU_E0410 | 50 |
| Nov 2020 | SG | MV | *Anthoxanthum odoratum* | 20_REU_E0412 | 50 |
| Nov 2020 | SG | MV | *Cenchrus clandestinus* | 20_REU_E0415 | 50 |
| Nov 2020 | SG | MV | *Carex boryana* | 20_REU_E0418 | 50 |
| Nov 2020 | SG | PC | *Juncus effusus* | 20_REU_E0502 | 50 |
| Nov 2020 | SG | PC | *Holcus lanatus* | 20_REU_E0505 | 50 |
| Nov 2020 | SG | PC | *Anthoxanthum odoratum* | 20_REU_E0509 | 50 |
| Nov 2020 | SG | PC | *Carex leporina* | 20_REU_E0510 | 50 |
| Nov 2020 | SG | PC | *Panicum juniperinum* | 20_REU_E0513 | 50 |
| Nov 2020 | SG | PC | *Festuca borbonica* | 20_REU_E0515 | 50 |
| Nov 2020 | SG | PC | *Costularia melicoides* | 20_REU_E0516 | 50 |
| Nov 2020 | SG | PC | *Costularia cadetii* | 20_REU_E0517 | 50 |
| Nov 2020 | SG | PC | *Carex boryana* | 20_REU_E0518 | 50 |
| Jan 2021 | AE | BP | *Eragrostis sp.* | 21_REU_E0101 | 50 |
| Jan 2021 | AE | BP | *Digitaria ciliaris* | 21_REU_E0102 | 50 |
| Jan 2021 | AE | BP | *Cyperus rotundus* | 21_REU_E0103 | 50 |
| Jan 2021 | AE | BP | *Paspalum dilatatum* | 21_REU_E0104 | 50 |
| Jan 2021 | AE | BP | *Dactyloctenium sp.* | 21_REU_E0105 | 50 |
| Jan 2021 | AE | BP | *Chloris gayana* | 21_REU_E0106 | 50 |
| Jan 2021 | AE | BP | *Megathyrsus maximus* | 21_REU_E0107 | 50 |
| Jan 2021 | AE | BP | *Melinis repens* | 21_REU_E0108 | 50 |
| Jan 2021 | AE | BP | *Cynodon dactylon* | 21_REU_E0109 | 50 |
| Jan 2021 | AE | BP | *Sorghum arundinaceum* | 21_REU_E0110 | 50 |
| Jan 2021 | AE | BP | *Cenchrus echinatus* | 21_REU_E0111 | 50 |
| Jan 2021 | AE | BP | *Eleusine indica* | 21_REU_E0112 | 50 |
| Jan 2021 | AE | BP | *Urochloa deflexa* | 21_REU_E0113 | 50 |
| Apr 2021 | CG | TR | *Cynodon dactylon* | 21_REU_E0201 | 50 |
| Apr 2021 | CG | TR | *Dactyloctenium sp.* | 21_REU_E0202 | 50 |
| Apr 2021 | CG | TR | *Heteropogon contortus* | 21_REU_E0203 | 50 |
| Apr 2021 | CG | TR | *Megathyrsus maximus* | 21_REU_E0204 | 50 |
| Apr 2021 | CG | PS | *Chloris barbata* | 21_REU_E0701 | 50 |
| Apr 2021 | CG | PS | *Cynodon dactylon* | 21_REU_E0702 | 50 |
| Apr 2021 | CG | PS | *Dactyloctenium sp.* | 21_REU_E0703 | 50 |
| Apr 2021 | CG | PS | *Heteropogon contortus* | 21_REU_E0704 | 50 |
| Apr 2021 | CG | PS | *Megathyrsus maximus* | 21_REU_E0705 | 50 |
| Apr 2021 | CG | PS | *Setaria pumila* | 21_REU_E0706 | 50 |
| Apr 2021 | CG | PS | *Urochloa panicoides* | 21_REU_E0707 | 50 |
| Apr 2021 | CG | PS | *Bothriochloa sp.* | 21_REU_E0708 | 50 |
| Apr 2021 | CG | PS | *Tragus mongolorum* | 21_REU_E0709 | 50 |
| Apr 2021 | CG | CH | *Aristida adscensionis* | 21_REU_E0601 | 50 |
| Apr 2021 | CG | CH | *Chloris barbata* | 21_REU_E0602 | 50 |
| Apr 2021 | CG | CH | *Cynodon dactylon* | 21_REU_E0603 | 50 |
| Apr 2021 | CG | CH | *Dactyloctenium sp.* | 21_REU_E0604 | 50 |
| Apr 2021 | CG | CH | *Digitaria ciliaris* | 21_REU_E0605 | 50 |
| Apr 2021 | CG | CH | *Eleusine indica* | 21_REU_E0606 | 50 |
| Apr 2021 | CG | CH | *Enneapogon cenchroides* | 21_REU_E0607 | 50 |
| Apr 2021 | CG | CH | *Heteropogon contortus* | 21_REU_E0608 | 50 |
| Apr 2021 | CG | CH | *Megathyrsus maximus* | 21_REU_E0609 | 50 |
| Apr 2021 | CG | CH | *Melinis repens* | 21_REU_E0610 | 50 |
| Apr 2021 | CG | CH | *Themeda quadrivalvis* | 21_REU_E0611 | 50 |
| Apr 2021 | CG | CH | *Urochloa panicoides* | 21_REU_E0612 | 50 |
| Apr 2021 | CG | CH | *Urochloa ramosa* | 21_REU_E0613 | 50 |
| Apr 2021 | AE | BP | *Stenotaphrum dimidiatum* | 21_REU_E0116 | 50 |
| Apr 2021 | AE | BP | *Cenchrus echinatus* | 21_REU_E0117 | 50 |
| Apr 2021 | AE | BP | *Chloris gayana* | 21_REU_E0118 | 50 |
| Apr 2021 | AE | BP | *Chrysopogon zizanioides* | 21_REU_E0119 | 50 |
| Apr 2021 | AE | BP | *Cynodon dactylon* | 21_REU_E0120 | 50 |
| Apr 2021 | AE | BP | *Dactyloctenium sp.* | 21_REU_E0121 | 50 |
| Apr 2021 | AE | BP | *Eleusine indica* | 21_REU_E0122 | 50 |
| Apr 2021 | AE | BP | *Eragrostis sp.* | 21_REU_E0123 | 50 |
| Apr 2021 | AE | BP | *Megathyrsus maximus* | 21_REU_E0124 | 50 |
| Apr 2021 | AE | BP | *Melinis repens* | 21_REU_E0125 | 50 |
| Apr 2021 | AE | BP | *Paspalum dilatatum* | 21_REU_E0126 | 50 |
| Apr 2021 | AE | BP | *Saccharum officinarum* | 21_REU_E0127 | 50 |
| Apr 2021 | AE | BP | *Sorghum arundinaceum* | 21_REU_E0128 | 50 |
| Apr 2021 | AE | BP | *Zea mays* | 21_REU_E0129 | 50 |
| Apr 2021 | AE | BP | *Urochloa panicoides* | 21_REU_E0130 | 50 |
| Apr 2021 | AE | CL | *Bromus catharticus* | 21_REU_E0801 | 50 |
| Apr 2021 | AE | CL | *Cenchrus purpureus* | 21_REU_E0802 | 50 |
| Apr 2021 | AE | CL | *Chloris gayana* | 21_REU_E0803 | 50 |
| Apr 2021 | AE | CL | *Digitaria radicosa* | 21_REU_E0804 | 50 |
| Apr 2021 | AE | CL | *Eleusine indica* | 21_REU_E0805 | 50 |
| Apr 2021 | AE | CL | *Megathyrsus maximus* | 21_REU_E0806 | 50 |
| Apr 2021 | AE | CL | *Urochloa decumbens* | 21_REU_E0807 | 50 |
| Apr 2021 | AE | CL | *Paspalum paniculatum* | 21_REU_E0808 | 50 |
| Apr 2021 | AE | CL | *Cenchrus purpureus* | 21_REU_E0809 | 50 |
| Apr 2021 | AE | CL | *Sporobolus africanus* | 21_REU_E0810 | 50 |
| Apr 2021 | AE | CL | *Sorghum arundinaceum* | 21_REU_E0811 | 50 |
| Apr 2021 | AE | CL | *Stenotaphrum dimidiatum* | 21_REU_E0812 | 50 |
| Apr 2021 | AE | CL | *Zea mays* | 21_REU_E0813 | 50 |
| Nov 2021 | CG | TR | *Bothriochloa sp.* | 21_REU_E0205 | 50 |
| Nov 2021 | CG | TR | *Chloris barbata* | 21_REU_E0206 | 50 |
| Nov 2021 | CG | TR | *Cynodon dactylon* | 21_REU_E0207 | 50 |
| Nov 2021 | CG | TR | *Dactyloctenium sp.* | 21_REU_E0208 | 15 |
| Nov 2021 | CG | TR | *Dichanthium aristatum* | 21_REU_E0209 | 50 |
| Nov 2021 | CG | TR | *Megathyrsus maximus* | 21_REU_E0210 | 50 |
| Nov 2021 | CG | PS | *Chloris barbata* | 21_REU_E0710 | 50 |
| Nov 2021 | CG | PS | *Cynodon dactylon* | 21_REU_E0711 | 50 |
| Nov 2021 | CG | PS | *Dichanthium aristatum* | 21_REU_E0712 | 50 |
| Nov 2021 | CG | PS | *Heteropogon contortus* | 21_REU_E0713 | 50 |
| Nov 2021 | CG | PS | *Fimbristylis cymosa* | 21_REU_E0714 | 50 |
| Nov 2021 | CG | PS | *Megathyrsus maximus* | 21_REU_E0715 | 50 |
| Nov 2021 | CG | CH | *Cynodon dactylon* | 21_REU_E0614 | 50 |
| Nov 2021 | CG | CH | *Dichanthium aristatum* | 21_REU_E0615 | 50 |
| Nov 2021 | CG | CH | *Enneapogon cenchroides* | 21_REU_E0616 | 50 |
| Nov 2021 | CG | CH | *Heteropogon contortus* | 21_REU_E0617 | 50 |
| Nov 2021 | CG | CH | *Megathyrsus maximus* | 21_REU_E0618 | 50 |
| Nov 2021 | AE | BP | *Bromus catharticus* | 21_REU_E0132 | 50 |
| Nov 2021 | AE | BP | *Cenchrus echinatus* | 21_REU_E0133 | 5 |
| Nov 2021 | AE | BP | *Chloris gayana* | 21_REU_E0134 | 50 |
| Nov 2021 | AE | BP | *Chrysopogon zizanioides* | 21_REU_E0135 | 50 |
| Nov 2021 | AE | BP | *Cynodon dactylon* | 21_REU_E0136 | 50 |
| Nov 2021 | AE | BP | *Cyperus rotundus* | 21_REU_E0137 | 50 |
| Nov 2021 | AE | BP | *Digitaria ciliaris* | 21_REU_E0138 | 50 |
| Nov 2021 | AE | BP | *Eleusine indica* | 21_REU_E0139 | 15 |
| Nov 2021 | AE | BP | *Eragrostis sp.* | 21_REU_E0140 | 50 |
| Nov 2021 | AE | BP | *Megathyrsus maximus* | 21_REU_E0141 | 50 |
| Nov 2021 | AE | BP | *Melinis repens* | 21_REU_E0142 | 50 |
| Nov 2021 | AE | BP | *Saccharum officinarum* | 21_REU_E0143 | 50 |
| Nov 2021 | AE | BP | *Sorghum arundinaceum* | 21_REU_E0144 | 50 |
| Nov 2021 | AE | BP | *Stenotaphrum dimidiatum* | 21_REU_E0145 | 50 |
| Nov 2021 | AE | BP | *Urochloa ramosa* | 21_REU_E0146 | 50 |
| Nov 2021 | AE | BP | *Zea mays* | 21_REU_E0147 | 50 |
| Nov 2021 | AE | LP | *Bromus catharticus* | 21_REU_E1001 | 50 |
| Nov 2021 | AE | LP | *Cynodon dactylon* | 21_REU_E1002 | 50 |
| Nov 2021 | AE | LP | *Eleusine indica* | 21_REU_E1003 | 30 |
| Nov 2021 | AE | LP | *Eragrostis sp.* | 21_REU_E1004 | 45 |
| Nov 2021 | AE | LP | *Megathyrsus maximus* | 21_REU_E1005 | 22 |
| Nov 2021 | AE | LP | *Melinis repens* | 21_REU_E1006 | 32 |
| Nov 2021 | AE | LP | *Saccharum officinarum* | 21_REU_E1007 | 50 |
| Nov 2021 | AE | LP | *Sorghum arundinaceum* | 21_REU_E1008 | 27 |
| Nov 2021 | AE | LP | *Chloris sp.* | 21_REU_E1009 | 50 |
| Nov 2021 | AE | LP | *Zea mays* | 21_REU_E1010 | 50 |
| Nov 2021 | AE | CL | *Bromus catharticus* | 21_REU_E0815 | 50 |
| Nov 2021 | AE | CL | *Cenchrus purpureus* | 21_REU_E0816 | 50 |
| Nov 2021 | AE | CL | *Chloris gayana* | 21_REU_E0817 | 50 |
| Nov 2021 | AE | CL | *Eragrostis sp.* | 21_REU_E0818 | 50 |
| Nov 2021 | AE | CL | *Urochloa decumbens* | 21_REU_E0819 | 50 |
| Nov 2021 | AE | CL | *Paspalum paniculatum* | 21_REU_E0820 | 50 |
| Nov 2021 | AE | CL | *Sporobolus africanus* | 21_REU_E0821 | 50 |
| Nov 2021 | AE | CL | *Stenotaphrum dimidiatum* | 21_REU_E0822 | 50 |
| Nov 2021 | AE | CL | *Cynodon sp.* | 21_REU_E0823 | 50 |
| Nov 2021 | SG | LE | *Bromus catharticus* | 21_REU_E0301 | 50 |
| Nov 2021 | SG | LE | *Cenchrus clandestinus* | 21_REU_E0302 | 50 |
| Nov 2021 | SG | LE | *Cenchrus purpureus* | 21_REU_E0303 | 50 |
| Nov 2021 | SG | LE | *Cynodon dactylon* | 21_REU_E0304 | 50 |
| Nov 2021 | SG | LE | *Cyperus aromaticus* | 21_REU_E0305 | 50 |
| Nov 2021 | SG | LE | *Holcus lanatus* | 21_REU_E0306 | 50 |
| Nov 2021 | SG | LE | *Setaria sp.* | 21_REU_E0307 | 50 |
| Nov 2021 | SG | LE | *Sporobolus africanus* | 21_REU_E0308 | 50 |
| Nov 2021 | SG | MV | *Anthoxanthum odoratum* | 21_REU_E0401 | 50 |
| Nov 2021 | SG | MV | *Carex leporina* | 21_REU_E0402 | 50 |
| Nov 2021 | SG | MV | *Cenchrus clandestinus* | 21_REU_E0403 | 50 |
| Nov 2021 | SG | MV | *Dactylis glomerata* | 21_REU_E0404 | 50 |
| Nov 2021 | SG | MV | *Holcus lanatus* | 21_REU_E0405 | 50 |
| Nov 2021 | SG | MV | *Juncus effusus* | 21_REU_E0406 | 50 |
| Nov 2021 | SG | PC | *Anthoxanthum odoratum* | 21_REU_E0501 | 50 |
| Nov 2021 | SG | PC | *Asterochaete nitens* | 21_REU_E0502 | 50 |
| Nov 2021 | SG | PC | *Carex boryana* | 21_REU_E0503 | 50 |
| Nov 2021 | SG | PC | *Carex pilulifera* | 21_REU_E0504 | 50 |
| Nov 2021 | SG | PC | *Festuca borbonica* | 21_REU_E0505 | 50 |
| Nov 2021 | SG | PC | *Holcus lanatus* | 21_REU_E0506 | 50 |
| Nov 2021 | SG | PC | *Juncus effusus* | 21_REU_E0507 | 50 |
| Nov 2021 | SG | PC | *Panicum juniperinum* | 21_REU_E0508 | 50 |
| Apr 2022 | CG | TR | *Chloris barbata* | 22_REU_E0201 | 50 |
| Apr 2022 | CG | TR | *Cynodon dactylon* | 22_REU_E0202 | 50 |
| Apr 2022 | CG | TR | *Dactyloctenium sp.* | 22_REU_E0203 | 50 |
| Apr 2022 | CG | TR | *Heteropogon contortus* | 22_REU_E0204 | 50 |
| Apr 2022 | CG | TR | *Megathyrsus maximus* | 22_REU_E0205 | 50 |
| Apr 2022 | CG | TR | *Stenotaphrum dimidiatum* | 22_REU_E0206 | 50 |
| Apr 2022 | CG | TR | *Urochloa panicoides* | 22_REU_E0207 | 50 |
| Apr 2022 | CG | PS | *Cenchrus clandestinus* | 22_REU_E0701 | 50 |
| Apr 2022 | CG | PS | *Chloris barbata* | 22_REU_E0702 | 50 |
| Apr 2022 | CG | PS | *Cynodon dactylon* | 22_REU_E0703 | 50 |
| Apr 2022 | CG | PS | *Dactyloctenium sp.* | 22_REU_E0704 | 50 |
| Apr 2022 | CG | PS | *Dichanthium aristatum* | 22_REU_E0705 | 50 |
| Apr 2022 | CG | PS | *Heteropogon contortus* | 22_REU_E0706 | 50 |
| Apr 2022 | CG | PS | *Fimbristylis cymosa* | 22_REU_E0707 | 50 |
| Apr 2022 | CG | PS | *Megathyrsus maximus* | 22_REU_E0708 | 50 |
| Apr 2022 | CG | PS | *Setaria pumila* | 22_REU_E0709 | 50 |
| Apr 2022 | CG | PS | *Tragus mongolorum* | 22_REU_E0710 | 50 |
| Apr 2022 | CG | PS | *Urochloa deflexa* | 22_REU_E0711 | 50 |
| Apr 2022 | CG | CH | *Aristida adscensionis* | 22_REU_E0601 | 50 |
| Apr 2022 | CG | CH | *Chloris barbata* | 22_REU_E0602 | 50 |
| Apr 2022 | CG | CH | *Chloris gayana* | 22_REU_E0603 | 50 |
| Apr 2022 | CG | CH | *Cynodon dactylon* | 22_REU_E0604 | 50 |
| Apr 2022 | CG | CH | *Dactyloctenium sp.* | 22_REU_E0605 | 50 |
| Apr 2022 | CG | CH | *Dichanthium aristatum* | 22_REU_E0606 | 50 |
| Apr 2022 | CG | CH | *Digitaria ciliaris* | 22_REU_E0607 | 50 |
| Apr 2022 | CG | CH | *Echinochloa colona* | 22_REU_E0608 | 50 |
| Apr 2022 | CG | CH | *Eleusine indica* | 22_REU_E0609 | 50 |
| Apr 2022 | CG | CH | *Enneapogon cenchroides* | 22_REU_E0610 | 50 |
| Apr 2022 | CG | CH | *Eragrostis sp.* | 22_REU_E0611 | 50 |
| Apr 2022 | CG | CH | *Heteropogon contortus* | 22_REU_E0612 | 50 |
| Apr 2022 | CG | CH | *Megathyrsus maximus* | 22_REU_E0613 | 50 |
| Apr 2022 | CG | CH | *Melinis repens* | 22_REU_E0614 | 50 |
| Apr 2022 | CG | CH | *Themeda quadrivalvis* | 22_REU_E0615 | 50 |
| Apr 2022 | CG | CH | *Urochloa deflexa* | 22_REU_E0616 | 50 |
| Apr 2022 | CG | CH | *Urochloa ramosa* | 22_REU_E0617 | 50 |
| Apr 2022 | AE | LM | *Cenchrus echinatus* | 22_REU_E1201 | 50 |
| Apr 2022 | AE | LM | *Chloris barbata* | 22_REU_E1202 | 50 |
| Apr 2022 | AE | LM | *Chloris pycnothrix* | 22_REU_E1203 | 50 |
| Apr 2022 | AE | LM | *Cynodon dactylon* | 22_REU_E1204 | 50 |
| Apr 2022 | AE | LM | *Dactyloctenium sp.* | 22_REU_E1205 | 50 |
| Apr 2022 | AE | LM | *Echinochloa colona* | 22_REU_E1206 | 50 |
| Apr 2022 | AE | LM | *Eleusine indica* | 22_REU_E1207 | 23 |
| Apr 2022 | AE | LM | *Eragrostis sp.* | 22_REU_E1208 | 50 |
| Apr 2022 | AE | LM | *Megathyrsus maximus* | 22_REU_E1209 | 50 |
| Apr 2022 | AE | LM | *Melinis repens* | 22_REU_E1210 | 50 |
| Apr 2022 | AE | LM | *Paspalum dilatatum* | 22_REU_E1211 | 50 |
| Apr 2022 | AE | LM | *Paspalum paniculatum* | 22_REU_E1212 | 50 |
| Apr 2022 | AE | LM | *Rottboellia cochinchinensis* | 22_REU_E1213 | 50 |
| Apr 2022 | AE | LM | *Saccharum officinarum* | 22_REU_E1214 | 50 |
| Apr 2022 | AE | LM | *Sorghum arundinaceum* | 22_REU_E1215 | 50 |
| Apr 2022 | AE | LM | *Sporobolus africanus* | 22_REU_E1216 | 50 |
| Apr 2022 | AE | LM | *Stenotaphrum dimidiatum* | 22_REU_E1217 | 50 |
| Apr 2022 | AE | LM | *Urochloa panicoides* | 22_REU_E1218 | 6 |
| Apr 2022 | AE | BP | *Cenchrus echinatus* | 22_REU_E0101 | 50 |
| Apr 2022 | AE | BP | *Chloris barbata* | 22_REU_E0102 | 50 |
| Apr 2022 | AE | BP | *Chloris gayana* | 22_REU_E0103 | 50 |
| Apr 2022 | AE | BP | *Chrysopogon zizanioides* | 22_REU_E0104 | 50 |
| Apr 2022 | AE | BP | *Cynodon dactylon* | 22_REU_E0105 | 50 |
| Apr 2022 | AE | BP | *Cyperus aromaticus* | 22_REU_E0106 | 50 |
| Apr 2022 | AE | BP | *Dactyloctenium sp.* | 22_REU_E0107 | 50 |
| Apr 2022 | AE | BP | *Digitaria ciliaris* | 22_REU_E0108 | 50 |
| Apr 2022 | AE | BP | *Eleusine indica* | 22_REU_E0109 | 50 |
| Apr 2022 | AE | BP | *Eragrostis sp.* | 22_REU_E0110 | 50 |
| Apr 2022 | AE | BP | *Megathyrsus maximus* | 22_REU_E0111 | 50 |
| Apr 2022 | AE | BP | *Melinis repens* | 22_REU_E0112 | 50 |
| Apr 2022 | AE | BP | *Paspalum dilatatum* | 22_REU_E0113 | 50 |
| Apr 2022 | AE | BP | *Paspalum paniculatum* | 22_REU_E0114 | 50 |
| Apr 2022 | AE | BP | *Pennisetum glaucum* | 22_REU_E0115 | 50 |
| Apr 2022 | AE | BP | *Saccharum officinarum* | 22_REU_E0116 | 50 |
| Apr 2022 | AE | BP | *Sorghum arundinaceum* | 22_REU_E0117 | 50 |
| Apr 2022 | AE | BP | *Sorghum bicolor* | 22_REU_E0118 | 50 |
| Apr 2022 | AE | BP | *Sporobolus africanus* | 22_REU_E0119 | 50 |
| Apr 2022 | AE | BP | *Stenotaphrum dimidiatum* | 22_REU_E0120 | 50 |
| Apr 2022 | AE | BP | *Urochloa panicoides* | 22_REU_E0121 | 50 |
| Apr 2022 | AE | BP | *Zea mays* | 22_REU_E0122 | 50 |
| Apr 2022 | AE | LP | *Cenchrus echinatus* | 22_REU_E1001 | 21 |
| Apr 2022 | AE | LP | *Chloris barbata* | 22_REU_E1002 | 50 |
| Apr 2022 | AE | LP | *Chloris gayana* | 22_REU_E1003 | 50 |
| Apr 2022 | AE | LP | *Chloris pycnothrix* | 22_REU_E1004 | 50 |
| Apr 2022 | AE | LP | *Cynodon dactylon* | 22_REU_E1005 | 50 |
| Apr 2022 | AE | LP | *Digitaria ciliaris* | 22_REU_E1006 | 50 |
| Apr 2022 | AE | LP | *Eleusine indica* | 22_REU_E1007 | 50 |
| Apr 2022 | AE | LP | *Eragrostis sp.* | 22_REU_E1008 | 50 |
| Apr 2022 | AE | LP | *Megathyrsus maximus* | 22_REU_E1009 | 50 |
| Apr 2022 | AE | LP | *Melinis repens* | 22_REU_E1010 | 50 |
| Apr 2022 | AE | LP | *Urochloa decumbens* | 22_REU_E1011 | 50 |
| Apr 2022 | AE | LP | *Saccharum officinarum* | 22_REU_E1012 | 50 |
| Apr 2022 | AE | LP | *Sporobolus africanus* | 22_REU_E1013 | 50 |
| Apr 2022 | AE | LP | *Sorghum arundinaceum* | 22_REU_E1014 | 50 |
| Apr 2022 | AE | LP | *Urochloa deflexa* | 22_REU_E1015 | 50 |
| Apr 2022 | AE | LP | *Urochloa panicoides* | 22_REU_E1016 | 50 |
| Apr 2022 | AE | LP | *Zea mays* | 22_REU_E1017 | 50 |
| Apr 2022 | AE | CL | *Bromus catharticus* | 22_REU_E0801 | 50 |
| Apr 2022 | AE | CL | *Cenchrus purpureus* | 22_REU_E0802 | 50 |
| Apr 2022 | AE | CL | *Chloris gayana* | 22_REU_E0803 | 50 |
| Apr 2022 | AE | CL | *Cyperus aromaticus* | 22_REU_E0804 | 50 |
| Apr 2022 | AE | CL | *Cyperus polystachyos* | 22_REU_E0805 | 50 |
| Apr 2022 | AE | CL | *Eleusine indica* | 22_REU_E0806 | 50 |
| Apr 2022 | AE | CL | *Eragrostis sp.* | 22_REU_E0807 | 50 |
| Apr 2022 | AE | CL | *Megathyrsus maximus* | 22_REU_E0808 | 50 |
| Apr 2022 | AE | CL | *Urochloa decumbens* | 22_REU_E0809 | 50 |
| Apr 2022 | AE | CL | *Paspalum notatum* | 22_REU_E0810 | 50 |
| Apr 2022 | AE | CL | *Cenchrus sp.* | 22_REU_E0811 | 50 |
| Apr 2022 | AE | CL | *Paspalum virgatum* | 22_REU_E0812 | 50 |
| Apr 2022 | AE | CL | *Sporobolus africanus* | 22_REU_E0813 | 50 |
| Apr 2022 | AE | CL | *Sorghum arundinaceum* | 22_REU_E0814 | 50 |
| Apr 2022 | AE | CL | *Stenotaphrum dimidiatum* | 22_REU_E0815 | 50 |
| Total |  |  | 61 species | 273 pools | 13341 |

**Supplementary Table 2. List of all *Mastrevirus* species used for phylogenetic analyses.** GenBank accession numbers correspond to the nucleotide sequences for complete genomes and amino acid sequences for the replication associated protein (Rep) and capsid protein (CP).

| **Mastreviruses** | **Complete** | **Rep** | **CP** |
| --- | --- | --- | --- |
| North American maize-associated mastrevirus | MZ852895 | ULE36141 | ULE36139 |
| sugar beet mastrevirus | EU034169 | ABV82713 | ABV82711 |
| Melinis repens associated virus | MK546380 | QDO73338 | QDO73337 |
| Mastrevirus sp. | MN203180 | QHB15159 | QHB15156 |
| barley dwarf virus | HF968647 | CCW72656 | CCW72655 |
| cotton mastrevirus | HE956706 | CCI88380 | CCI88379 |
| maize streak virus | NC_001346 | YP_009154763 | YP_009154762 |
| chloris striate mosaic virus | NC_001466 | NP_597785 | NP_040952 |
| Digitaria streak virus | NC_001478 | NP_040965 | NP_040963 |
| Panicum streak virus | NC_001647 | NP_042590 | NP_042589 |
| sugarcane streak Egypt virus | NC_001868 | NP_045945 | NP_045943 |
| wheat dwarf virus | NC_003326 | NP_542349 | NP_542348 |
| bean yellow dwarf virus | NC_003493 | NP_612221 | NP_612220 |
| sugarcane streak virus | NC_003744 | NP_620492 | NP_620491 |
| tobacco yellow dwarf virus | NC_003822 | NP_620727 | NP_620725 |
| sugarcane streak Reunion virus | NC_004755 | NP_840053 | NP_840051 |
| Eragrostis streak virus | NC_010352 | YP_001686794 | YP_001686793 |
| Urochloa streak virus | NC_010797 | YP_001941155 | YP_001941154 |
| oat dwarf virus | NC_010799 | YP_001941162 | YP_001941161 |
| chickpea chlorotic dwarf virus | NC_011058 | YP_002014713 | YP_002014711 |
| Saccharum streak virus | NC_013464 | YP_003288768 | YP_003288767 |
| Digitaria didactyla striate mosaic virus | NC_014547 | YP_003915159 | YP_003915158 |
| chickpea redleaf virus | NC_014739 | YP_004046663 | YP_004046661 |
| chickpea chlorosis virus | NC_014740 | YP_004046667 | YP_004046665 |
| Bromus catharticus striate mosaic virus | NC_014822 | YP_004089627 | YP_004089626 |
| Eragrostis minor streak virus | NC_015553 | YP_004465364 | YP_004465363 |
| wheat dwarf India virus | NC_017828 | YP_006273070 | YP_006273069 |
| maize streak Reunion virus | NC_017917 | YP_006331073 | YP_006331072 |
| Paspalum striate mosaic virus | NC_018530 | YP_006659974 | YP_006659973 |
| Paspalum dilatatum striate mosaic virus | NC_018576 | YP_006666523 | YP_006666522 |
| Sporobolus striate mosaic virus 1 | NC_018577 | YP_006666527 | YP_006666526 |
| Sporobolus striate mosaic virus 2 | NC_018578 | YP_006666531 | YP_006666530 |
| Digitaria ciliaris striate mosaic virus | NC_018579 | YP_006666535 | YP_006666534 |
| dragonfly-associated mastrevirus | NC_019497 | YP_007004038 | YP_007004037 |
| chickpea chlorosis Australia virus | NC_022131 | YP_008472704 | YP_008472703 |
| Axonopus compressus streak virus | NC_023864 | YP_009021763 | YP_009021762 |
| sugarcane white streak virus | NC_023989 | YP_009026388 | YP_009026387 |
| chickpea yellow dwarf virus | NC_025475 | YP_009104363 | YP_009104362 |
| switchgrass mosaic-associated virus 1 | NC_025834 | YP_009111310 | YP_009111308 |
| sugarcane chlorotic streak virus | NC_032004 | YP_009325925 | YP_009325924 |
| sweet potato symptomless virus 1 | NC_034630 | YP_009362982 | YP_009362981 |
| chickpea yellows virus | NC_038478 | YP_009506577 | YP_009506576 |
| rice latent virus 2 | NC_040537 | YP_009551800 | YP_009551799 |
| maize striate mosaic virus | NC_040541 | YP_009551897 | YP_009551896 |
| rice latent virus 1 | NC_040792 | YP_009553475 | YP_009553474 |
| maize streak dwarfing virus | NC_055519 | YP_010087228 | YP_010087227 |
| Eleusine indica associated virus | NC_055572 | YP_010087740 | YP_010087739 |
| Sorghum arundinaceum associated virus | NC_055573 | YP_010087743 | YP_010087742 |
| chickpea redleaf virus 2 | NC_055579 | YP_010087772 | YP_010087771 |

**Supplementary Table 3. List of all the CRESS DNA viruses detected during the study.**

| **Survey** | **Type of Site** | **Site** | **Pool_Id** | **Host Specie** | **Virus** | **Contig size (nt)** | **Accession Number** | **Closest**  **species** | **Sequence identity** |
| --- | --- | --- | --- | --- | --- | --- | --- | --- | --- |
| Nov 2020 | AE | BP | 20_REU_E0109 | *Cynodon dactylon* | *Genomovirus* | 2,178 | PQ513396 | Genomoviridae sp. (NC_076327.1) | 95.56% |
| Jan 2021 | AE | BP | 21_REU_E0101 | *Eragrostis sp.* | *Genomovirus* | 2,192 | PQ513397 | Genomoviridae sp. (NC_076327.1) | 78.94% |
| Jan 2021 | AE | BP | 21_REU_E0101 | *Eragrostis sp.* | *Genomovirus* | 2,179 | PQ513398 | Plant associated genomovirus 2 (MH939413.1) | 96.33% |
| Apr 2021 | CG | CH | 21_REU_E0603 | *Cynodon dactylon* | *Genomovirus* | 2,164 | PQ513411 | Molossus molossus associated gemykibivirus 3 (OL704843.1) | 94.63% |
| Apr 2021 | CG | CH | 21_REU_E0605 | *Digitaria ciliaris* | *Genomovirus* | 2,159 | PQ513412 | Mute swan feces associated gemycircularvirus 1 (MW588093.1) | 88.58% |
| Apr 2021 | CG | PS | 21_REU_E0701 | *Chloris barbata* | *Genomovirus* | 1,700 | PQ513413 | Genomoviridae sp.  (OQ198261.1) | 91.89% |
| Apr 2021 | CG | PS | 21_REU_E0702 | *Cynodon dactylon* | *Genomovirus* | 2,909 | PQ513414 | Alces alces faeces associated circular virus (NC_040327.1) | 67.55% |
| Apr 2021 | CG | PS | 21_REU_E0702 | *Cynodon dactylon* | *Genomovirus* | 2,178 | PQ513415 | Red panda feces-associated genomovirus (MZ556214.1) | 83.72% |
| Apr 2021 | CG | PS | 22_REU_E0705 | *Dichanthium aristatum* | *Genomovirus* | 1,360 | PQ513417 | Genomoviridae sp.  (OR370203.1) | 83.94% |
| Apr 2021 | AE | BP | 21_REU_E0116 | *Stenotaphrum*  *dimidiatum* | *Genomovirus* | 2,194 | PQ513399 | Plant associated genomovirus 2 (MH939366.1) | 98.52% |
| Apr 2021 | AE | BP | 21_REU_E0118 | *Chloris gayana* | *Genomovirus* | 2,270 | PQ513400 | Genomoviridae sp.  (OM892306.1) | 86.17% |
| Apr 2021 | AE | BP | 21_REU_E0121 | *Dactyloctenium sp.* | *Genomovirus* | 973 | PQ513401 | Red panda feces-associated gemycircularvirus (MZ556166.1) | 95.53% |
| Apr 2021 | AE | BP | 21_REU_E0121 | *Dactyloctenium sp.* | *Genomovirus* | 2,207 | PQ513402 | Genomoviridae sp. (NC_076327.1) | 77.88% |
| Apr 2021 | AE | BP | 21_REU_E0121 | *Dactyloctenium sp.* | *Genomovirus* | 1,671 | PQ513403 | Genomoviridae sp.  (MN928914.1) | 96.90% |
| Apr 2021 | AE | BP | 21_REU_E0128 | *Sorghum*  *arundinaceum* | Sorghum mastrevirus associated  alphasatellite | 1,535 | PQ513404 | Sorghum mastrevirus associated alphasatellite (NC_076478.1) | 98.37% |
| Nov 2021 | AE | BP | 21_REU_E0134 | *Chloris gayana* | *Genomovirus* | 1,845 | PQ513405 | Plant associated genomovirus 2 (MH939412.1) | 90.72% |
| Nov 2021 | AE | BP | 21_REU_E0134 | *Chloris gayana* | *Genomovirus* | 2,049 | PQ513406 | Plant associated genomovirus 2 (MH939412.1) | 90.44% |
| Nov 2021 | AE | BP | 21_REU_E0136 | *Cynodon dactylon* | *Genomovirus* | 2,216 | PQ513407 | Gemycircularvirus (MW183036.1) | 91.32% |
| Nov 2021 | AE | BP | 21_REU_E0136 | *Cynodon dactylon* | *Genomovirus* | 2,212 | PQ513408 | Genomoviridae sp. (MW678953.1) | 89.93% |
| Nov 2021 | AE | BP | 21_REU_E0144 | *Sorghum*  *arundinaceum* | Sorghum mastrevirus associated  alphasatellite | 1,538 | PQ513410 | Sorghum mastrevirus associated alphasatellite (NC_076478.1) | 98.83% |
| Apr 2022 | AE | BP | 22_REU_E0117 | *Sorghum*  *arundinaceum* | Sorghum mastrevirus associated  alphasatellite | 1,535 | PQ513416 | Sorghum mastrevirus associated alphasatellite (NC_076478.1) | 98.37% |
| Apr 2022 | AE | CL | 22_REU_E0807 | *Eragrostis sp.* | *Genomovirus* | 1,389 | PQ513418 | Genomoviridae sp.  (MT138093.1) | 100.00% |
| Apr 2022 | AE | CL | 22_REU_E0807 | *Eragrostis sp.* | *Genomovirus* | 1,144 | PQ513419 | Genomoviridae sp.  (MT138093.1) | 100.00% |
| Apr 2022 | AE | LP | 22_REU_E1017 | *Zea mays* | *Genomovirus* | 2,184 | PQ513420 | Genomoviridae sp. (NC_076327.1) | 78.78% |
| Apr 2022 | AE | LM | 22_REU_E1208 | *Eragrostis sp.* | *Genomovirus* | 2,198 | PQ513421 | Genomoviridae sp.  (PP744644.1) | 88.73% |

**Supplementary Table 4. Summary of putative new *Mastrevirus* species.** Sequence identity is based on complete genomes and is calculated from nucleotide sequences.

| **Common name** | **Acronyme** | **Tentative species name** | **Lenght (nt)** | **MP** | **CP** | **Rep** | **RepA** | **Nonanucleotide** | **Closest species** | **Sequence identity** |
| --- | --- | --- | --- | --- | --- | --- | --- | --- | --- | --- |
| Cenchrus echinatus associated virus | CEAV | *Mastrevirus cenchri* | 2,809-2,811 | 102-472 | 682-1212 | 2,619-1,954 / 1,844-1,551 | 2,619-1954 | TAATATTAC | WDIV (MN273439) | 58.6% |
| Cenchrus purpureus mild streak virus | CPMSV | *Mastrevirus purpurei* | 2,808 | 184-495 | 539-1,078 | 2,647-2,231 / 1,926-1,480 | 2,647-2,228 | TAATATTAC | SAAV (MK546381) | 57.9% |
| Urochloa decumbens associated virus | UDAV | *Mastrevirus*  *urochloareunionense* | 2,837 | 169-468 | 530-1,282 | 2,668-1,823 / 1,736-1,452 | 2,668-1766 | TAATATTGC | ACSV (KJ437671) | 72.3% |
| sub-genomic  mastrevirus | DefMS | *NA* | 1,505-1,508 | 103-426 | 415-1069 | *NA* | *NA* | TAATATTAC | NAMaV (MZ852895) | 57.2% |

**Supplementary Table 5. Summary of the sequenced *Mastrevirus* species in each plant pool.** MSV-F: maize streak virus strain F; USV: Urochloa streak virus; MSV-B: maize streak virus strain B; CEAV: Cenchrus echinatus associated virus; MSRV: maize streak Reunion virus; SSRV: sugarcane streak Reunion virus; PanSV-J: Panicum streak virus strain J; EIAV: Eleusine indica associated virus; SWSV: sugarcane white streak virus; SAAV-B: Sorghum arundinaceum associated virus strain B; UDAV: Urochloa decumbens associated virus; PanSV-C: Panicum streak virus strain C; DefMS: defective Mastrevirus species; CPMSV: Cenchrus purpureus mild streak virus; SAAV-A: Sorghum arundinaceum associated virus strain A.

| **Survey** | **Type of Site** | **Site** | **Pool Id** | **Host Species** | **Virus Species** | **Sequencing Procedure** | **Restriction Site** | **Accession Number** |
| --- | --- | --- | --- | --- | --- | --- | --- | --- |
| Jan 2021 | AE | BP | 21_REU_E0111 | *Cenchrus echinatus* | MSV-F | RCA-MinION | *NA* | PQ434707 |
| Jan 2021 | AE | BP | 21_REU_E0111 | *Cenchrus echinatus* | USV | RCA-MinION | *NA* | PQ434708 |
| Jan 2021 | AE | BP | 21_REU_E0111 | *Cenchrus echinatus* | USV | RCA-RFLP | *Kpn*I | PQ513427 |
| Jan 2021 | AE | BP | 21_REU_E0111 | *Cenchrus echinatus* | USV | RCA-RFLP | *Kpn*I | PQ513428 |
| Jan 2021 | AE | BP | 21_REU_E0111 | *Cenchrus echinatus* | USV | RCA-RFLP | *Kpn*I | PQ513429 |
| Apr 2021 | CG | TR | 21_REU_E0203 | *Heteropogon contortus* | USV | RCA-MinION | *NA* | PQ434724 |
| Apr 2021 | CG | CH | 21_REU_E0612 | *Urochloa panicoides* | MSV-B | RCA-MinION | *NA* | PQ434725 |
| Apr 2021 | CG | CH | 21_REU_E0612 | *Urochloa panicoides* | USV | RCA-MinION | *NA* | PQ434726 |
| Apr 2021 | AE | BP | 21_REU_E0116 | *Stenotaphrum dimidiatum* | USV | RCA-MinION | *NA* | PQ434709 |
| Apr 2021 | AE | BP | 21_REU_E0117 | *Cenchrus echinatus* | CEAV | RCA-MinION | *NA* | PQ434710 |
| Apr 2021 | AE | BP | 21_REU_E0117 | *Cenchrus echinatus* | MSRV | RCA-MinION | *NA* | PQ434711 |
| Apr 2021 | AE | BP | 21_REU_E0117 | *Cenchrus echinatus* | MSV-B | RCA-MinION | *NA* | PQ434712 |
| Apr 2021 | AE | BP | 21_REU_E0117 | *Cenchrus echinatus* | SSRV | RCA-MinION | *NA* | PQ434713 |
| Apr 2021 | AE | BP | 21_REU_E0117 | *Cenchrus echinatus* | USV | RCA-MinION | *NA* | PQ434714 |
| Apr 2021 | AE | BP | 21_REU_E0119 | *Chrysopogon zizanioides* | MSRV | RCA-MinION | *NA* | PQ434715 |
| Apr 2021 | AE | BP | 21_REU_E0120 | *Cynodon dactylon* | PanSV-J | RCA-MinION | *NA* | PQ434716 |
| Apr 2021 | AE | BP | 21_REU_E0122 | *Eleusine indica* | EIAV | RCA-MinION | *NA* | PQ434717 |
| Apr 2021 | AE | BP | 21_REU_E0124 | *Megathyrsus maximus* | PanSV-J | RCA-MinION | *NA* | PQ434718 |
| Apr 2021 | AE | BP | 21_REU_E0127 | *Saccharum spp.* | SWSV | RCA-MinION | *NA* | PQ434719 |
| Apr 2021 | AE | BP | 21_REU_E0128 | *Sorghum arundinaceum* | SAAV-B | RCA-MinION | *NA* | PQ434720 |
| Apr 2021 | AE | BP | 21_REU_E0130 | *Urochloa panicoides* | USV | RCA-MinION | *NA* | PQ434721 |
| Apr 2021 | AE | CL | 21_REU_E0807 | *Urochloa decumbens* | UDAV | RCA-MinION | *NA* | PQ434728 |
| Apr 2021 | AE | CL | 21_REU_E0807 | *Urochloa decumbens* | UDAV | RCA-RFLP | *Xba*I | OQ451139 |
| Apr 2021 | AE | CL | 21_REU_E0807 | *Urochloa decumbens* | UDAV | RCA-RFLP | *Xba*I | OQ451140 |
| Apr 2021 | AE | CL | 21_REU_E0807 | *Urochloa decumbens* | UDAV | RCA-RFLP | *Xba*I | OQ451141 |
| Apr 2021 | AE | CL | 21_REU_E0807 | *Urochloa decumbens* | UDAV | RCA-RFLP | *Xba*I | OQ451142 |
| Apr 2021 | AE | CL | 21_REU_E0821 | *Sporobolus africanus* | MSV-B | RCA-MinION | *NA* | PQ434731 |
| Nov 2021 | CG | PS | 21_REU_E0715 | *Megathyrsus maximus* | PanSV-C | RCA-MinION | *NA* | PQ434727 |
| Nov 2021 | AE | BP | 21_REU_E0138 | *Digitaria ciliaris* | MSV-B | RCA-MinION | *NA* | PQ434722 |
| Nov 2021 | AE | BP | 21_REU_E0143 | *Saccharum spp.* | DefMS | RCA-MinION | *NA* | PQ513409 |
| Nov 2021 | AE | BP | 21_REU_E0143 | *Saccharum spp.* | DefMS | RCA-RFLP | *Nco*I | PQ513423 |
| Nov 2021 | AE | BP | 21_REU_E0143 | *Saccharum spp.* | DefMS | RCA-RFLP | *Nco*I | PQ513424 |
| Nov 2021 | AE | BP | 21_REU_E0143 | *Saccharum spp.* | DefMS | RCA-RFLP | *Nco*I | PQ513425 |
| Nov 2021 | AE | BP | 21_REU_E0143 | *Saccharum spp.* | DefMS | RCA-RFLP | *Nco*I | PQ513426 |
| Nov 2021 | AE | BP | 21_REU_E0143 | *Saccharum spp.* | SWSV | RCA-MinION | *NA* | PQ434723 |
| Nov 2021 | AE | CL | 21_REU_E0816 | *Cenchrus purpureus* | CPMSV | RCA-MinION | *NA* | PQ434729 |
| Nov 2021 | AE | CL | 21_REU_E0816 | *Cenchrus purpureus* | CPMSV | RCA-RFLP | *Sal*I | OQ451138 |
| Nov 2021 | AE | CL | 21_REU_E0819 | *Urochloa decumbens* | UDAV | RCA-MinION | *NA* | PQ434730 |
| Nov 2021 | AE | CL | 21_REU_E0821 | *Sporobolus africanus* | MSV-B | RCA-MinION | *NA* | PQ434731 |
| Nov 2021 | AE | LP | 21_REU_E1007 | *Saccharum spp.* | SWSV | RCA-MinION | *NA* | PQ434732 |
| Nov 2021 | AE | LP | 21_REU_E1008 | *Sorghum arundinaceum* | SAAV-A | RCA-MinION | *NA* | PQ434733 |
| Apr 2022 | CG | CH | 22_REU_E0607 | *Digitaria ciliaris* | MSV-B | RCA-MinION | *NA* | PQ434749 |
| Apr 2022 | CG | CH | 22_REU_E0617 | *Urochloa ramosa* | USV | RCA-MinION | *NA* | PQ434750 |
| Apr 2022 | CG | PS | 22_REU_E0708 | *Megathyrsus maximus* | PanSV-C | RCA-MinION | *NA* | PQ434751 |
| Apr 2022 | CG | PS | 22_REU_E0711 | *Urochloa deflexa* | PanSV-C | RCA-MinION | *NA* | PQ434752 |
| Apr 2022 | CG | PS | 22_REU_E0711 | *Urochloa deflexa* | USV | RCA-MinION | *NA* | PQ434753 |
| Apr 2022 | AE | BP | 22_REU_E0101 | *Cenchrus echinatus* | CEAV | RCA-MinION | *NA* | PQ434734 |
| Apr 2022 | AE | BP | 22_REU_E0101 | *Cenchrus echinatus* | MSRV | RCA-MinION | *NA* | PQ434735 |
| Apr 2022 | AE | BP | 22_REU_E0101 | *Cenchrus echinatus* | MSV-B | RCA-MinION | *NA* | PQ434736 |
| Apr 2022 | AE | BP | 22_REU_E0101 | *Cenchrus echinatus* | SSRV | RCA-MinION | *NA* | PQ434737 |
| Apr 2022 | AE | BP | 22_REU_E0101 | *Cenchrus echinatus* | USV | RCA-MinION | *NA* | PQ434738 |
| Apr 2022 | AE | BP | 22_REU_E0103 | *Chloris gayana* | MSRV | RCA-MinION | *NA* | PQ434739 |
| Apr 2022 | AE | BP | 22_REU_E0105 | *Cynodon dactylon* | MSRV | RCA-MinION | *NA* | PQ434740 |
| Apr 2022 | AE | BP | 22_REU_E0109 | *Eleusine indica* | EIAV | RCA-MinION | *NA* | PQ434741 |
| Apr 2022 | AE | BP | 22_REU_E0111 | *Megathyrsus maximus* | PanSV-J | RCA-MinION | *NA* | PQ434742 |
| Apr 2022 | AE | BP | 22_REU_E0116 | *Saccharum spp.* | SWSV | RCA-MinION | *NA* | PQ434743 |
| Apr 2022 | AE | BP | 22_REU_E0117 | *Sorghum arundinaceum* | SAAV-A | RCA-MinION | *NA* | PQ434744 |
| Apr 2022 | AE | BP | 22_REU_E0118 | *Sorghum bicolor* | SAAV-B | RCA-MinION | *NA* | PQ434745 |
| Apr 2022 | AE | BP | 22_REU_E0121 | *Urochloa panicoides* | MSV-B | RCA-MinION | *NA* | PQ434746 |
| Apr 2022 | AE | BP | 22_REU_E0121 | *Urochloa panicoides* | USV | RCA-MinION | *NA* | PQ434747 |
| Apr 2022 | AE | BP | 22_REU_E0122 | *Zea mays* | MSRV | RCA-MinION | *NA* | PQ434748 |
| Apr 2022 | AE | CL | 22_REU_E0809 | *Urochloa decumbens* | UDAV | RCA-MinION | *NA* | PQ434754 |
| Apr 2022 | AE | LP | 22_REU_E1007 | *Eleusine indica* | EIAV | RCA-MinION | *NA* | PQ434755 |
| Apr 2022 | AE | LP | 22_REU_E1014 | *Sorghum arundinaceum* | SAAV-A | RCA-MinION | *NA* | PQ434756 |
| Apr 2022 | AE | LP | 22_REU_E1016 | *Urochloa panicoides* | MSV-B | RCA-MinION | *NA* | PQ434757 |
| Apr 2022 | AE | LP | 22_REU_E1017 | *Zea mays* | MSRV | RCA-MinION | *NA* | PQ434758 |
| Apr 2022 | AE | LM | 22_REU_E1201 | *Cenchrus echinatus* | MSRV | RCA-MinION | *NA* | PQ434759 |
| Apr 2022 | AE | LM | 22_REU_E1201 | *Cenchrus echinatus* | MSV-B | RCA-MinION | *NA* | PQ434760 |
| Apr 2022 | AE | LM | 22_REU_E1201 | *Cenchrus echinatus* | PanSV-J | RCA-MinION | *NA* | PQ434761 |
| Apr 2022 | AE | LM | 22_REU_E1202 | *Chloris barbata* | EIAV | RCA-MinION | *NA* | PQ434762 |
| Apr 2022 | AE | LM | 22_REU_E1202 | *Chloris barbata* | MSRV | RCA-MinION | *NA* | PQ434763 |
| Apr 2022 | AE | LM | 22_REU_E1207 | *Eleusine indica* | EIAV | RCA-MinION | *NA* | PQ434764 |
| Apr 2022 | AE | LM | 22_REU_E1207 | *Eleusine indica* | MSV-B | RCA-MinION | *NA* | PQ434765 |
| Apr 2022 | AE | LM | 22_REU_E1209 | *Megathyrsus maximus* | PanSV-J | RCA-MinION | *NA* | PQ434766 |
| Apr 2022 | AE | LM | 22_REU_E1209 | *Megathyrsus maximus* | SWSV | RCA-MinION | *NA* | PQ434767 |
| Apr 2022 | AE | LM | 22_REU_E1210 | *Melinis repens* | PanSV-J | RCA-MinION | *NA* | PQ434768 |
| Apr 2022 | AE | LM | 22_REU_E1213 | *Rottboellia cochinchinensis* | MSRV | RCA-MinION | *NA* | PQ434769 |
| Apr 2022 | AE | LM | 22_REU_E1213 | *Rottboellia cochinchinensis* | PanSV-J | RCA-MinION | *NA* | PQ434770 |
| Apr 2022 | AE | LM | 22_REU_E1214 | *Saccharum spp.* | DefMS | RCA-MinION | *NA* | PQ513422 |
| Apr 2022 | AE | LM | 22_REU_E1215 | *Sorghum arundinaceum* | SAAV-A | RCA-MinION | *NA* | PQ434771 |

**Supplementary Table 6. Summary of all the plant species.** Origin was determined for La Réunion region using the Conservatoire Botanique National de Mascarin botanical database (https://mascarine.cbnm.org/). Origin: Native or Introduced species; crop status: Uncultivated or Cultivated; life cycle: Annual, Perennial and Annual/Perennial.

| **Host Family** | **Host Tribe** | **Host Genus** | **Host Species** | **Origin** | **Crop Status** | **Life Cycle** |
| --- | --- | --- | --- | --- | --- | --- |
| Cyperaceae | Abildgaardieae | *Fimbristylis* | *Fimbristylis cymosa* | Native | Uncultivated | Perennial |
| Cyperaceae | Cariceae | *Carex* | *Carex boryana* | Native | Uncultivated | Perennial |
| Cyperaceae | Cariceae | *Carex* | *Carex leporina* | Introduced | Uncultivated | Perennial |
| Cyperaceae | Cariceae | *Carex* | *Carex pilulifera* | Introduced | Uncultivated | Perennial |
| Cyperaceae | Cypereae | *Cyperus* | *Cyperus aromaticus* | Native | Uncultivated | Perennial |
| Cyperaceae | Cypereae | *Cyperus* | *Cyperus polystachyos* | Native | Uncultivated | Perennial |
| Cyperaceae | Cypereae | *Cyperus* | *Cyperus rotundus* | Introduced | Uncultivated | Perennial |
| Cyperaceae | Schoeneae | *Asterochaete* | *Asterochaete nitens* | Native | Uncultivated | Perennial |
| Cyperaceae | Schoeneae | *Costularia* | *Costularia cadetii* | Native | Uncultivated | Perennial |
| Cyperaceae | Schoeneae | *Costularia* | *Costularia melicoides* | Native | Uncultivated | Perennial |
| Juncaceae | Juncus | *Juncus* | *Juncus effusus* | Native | Uncultivated | Perennial |
| Poaceae | Andropogoneae | *Bothriochloa* | *Bothriochloa sp.* | Native | Uncultivated | Annual |
| Poaceae | Andropogoneae | *Chrysopogon* | *Chrysopogon zizanioides* | Introduced | Cultivated | Perennial |
| Poaceae | Andropogoneae | *Dichanthium* | *Dichanthium aristatum* | Introduced | Uncultivated | Perennial |
| Poaceae | Andropogoneae | *Heteropogon* | *Heteropogon contortus* | Native | Uncultivated | Perennial |
| Poaceae | Andropogoneae | *Pennisetum* | *Pennisetum glaucum* | Introduced | Cultivated | Annual |
| Poaceae | Andropogoneae | *Rottboellia* | *Rottboellia cochinchinensis* | Introduced | Uncultivated | Annual |
| Poaceae | Andropogoneae | *Saccharum* | *Saccharum spp.* | Introduced | Cultivated | Perennial |
| Poaceae | Andropogoneae | *Sorghum* | *Sorghum arundinaceum* | Introduced | Uncultivated | Annual/Perennial |
| Poaceae | Andropogoneae | *Sorghum* | *Sorghum bicolor* | Introduced | Cultivated | Annual |
| Poaceae | Andropogoneae | *Themada* | *Themeda quadrivalvis* | Introduced | Uncultivated | Annual |
| Poaceae | Andropogoneae | *Zea* | *Zea mays* | Introduced | Cultivated | Annual |
| Poaceae | Aristideae | *Aristida* | *Aristida adscensionis* | Introduced | Uncultivated | Annual |
| Poaceae | Bromeae | *Bromus* | *Bromus catharticus* | Introduced | Uncultivated | Annual |
| Poaceae | Cynodonteae | *Chloris* | *Chloris barbata* | Introduced | Uncultivated | Perennial |
| Poaceae | Cynodonteae | *Chloris* | *Chloris gayana* | Introduced | Uncultivated | Perennial |
| Poaceae | Cynodonteae | *Chloris* | *Chloris pycnothrix* | Introduced | Uncultivated | Annual |
| Poaceae | Cynodonteae | *Chloris* | *Chloris sp.* | Introduced | Uncultivated | NA |
| Poaceae | Cynodonteae | *Cynodon* | *Cynodon dactylon* | Native | Uncultivated | Perennial |
| Poaceae | Cynodonteae | *Cynodon* | *Cynodon sp.* | Introduced | Uncultivated | Perennial |
| Poaceae | Cynodonteae | *Dactyloctenium* | *Dactyloctenium sp.* | Native | Uncultivated | Annual |
| Poaceae | Cynodonteae | *Eleusine* | *Eleusine indica* | Introduced | Uncultivated | Annual |
| Poaceae | Cynodonteae | *Tragus* | *Tragus mongolorum* | Introduced | Uncultivated | Annual |
| Poaceae | Eragrostideae | *Enneapogon* | *Enneapogon cenchroides* | Introduced | Uncultivated | Annual |
| Poaceae | Eragrostideae | *Eragrostis* | *Eragrostis sp.* | Introduced | Uncultivated | Annual/Perennial |
| Poaceae | Paniceae | *Cenchrus* | *Cenchrus clandestinus* | Introduced | Uncultivated | Perennial |
| Poaceae | Paniceae | *Cenchrus* | *Cenchrus echinatus* | Introduced | Uncultivated | Annual |
| Poaceae | Paniceae | *Cenchrus* | *Cenchrus purpureus* | Introduced | Cultivated | Perennial |
| Poaceae | Paniceae | *Cenchrus* | *Cenchrus sp.* | Introduced | Uncultivated | Perennial |
| Poaceae | Paniceae | *Digitaria* | *Digitaria ciliaris* | Introduced | Uncultivated | Annual |
| Poaceae | Paniceae | *Digitaria* | *Digitaria radicosa* | Introduced | Uncultivated | Annual |
| Poaceae | Paniceae | *Echinochloa* | *Echinochloa colona* | Introduced | Uncultivated | Annual |
| Poaceae | Paniceae | *Megathyrsus* | *Megathyrsus maximus* | Introduced | Uncultivated | Perennial |
| Poaceae | Paniceae | *Melinis* | *Melinis repens* | Introduced | Uncultivated | Annual/Perennial |
| Poaceae | Paniceae | *Panicum* | *Panicum juniperinum* | Native | Uncultivated | Perennial |
| Poaceae | Paniceae | *Setaria* | *Setaria pumila* | Introduced | Uncultivated | Annual |
| Poaceae | Paniceae | *Setaria* | *Setaria sp.* | Introduced | Uncultivated | Annual |
| Poaceae | Paniceae | *Stenotaphrum* | *Stenotaphrum dimidiatum* | Introduced | Uncultivated | Perennial |
| Poaceae | Paniceae | *Urochloa* | *Urochloa decumbens* | Introduced | Uncultivated | Annual |
| Poaceae | Paniceae | *Urochloa* | *Urochloa deflexa* | Introduced | Uncultivated | Annual |
| Poaceae | Paniceae | *Urochloa* | *Urochloa panicoides* | Introduced | Uncultivated | Annual |
| Poaceae | Paniceae | *Urochloa* | *Urochloa ramosa* | Introduced | Uncultivated | Annual |
| Poaceae | Paspaleae | *Paspalum* | *Paspalum dilatatum* | Introduced | Uncultivated | Perennial |
| Poaceae | Paspaleae | *Paspalum* | *Paspalum notatum* | Introduced | Uncultivated | Perennial |
| Poaceae | Paspaleae | *Paspalum* | *Paspalum paniculatum* | Introduced | Uncultivated | Perennial |
| Poaceae | Paspaleae | *Paspalum* | *Paspalum virgatum* | Introduced | Uncultivated | Perennial |
| Poaceae | Poeae | *Anthoxanthum* | *Anthoxanthum odoratum* | Introduced | Uncultivated | Perennial |
| Poaceae | Poeae | *Dactylis* | *Dactylis glomerata* | Introduced | Uncultivated | Perennial |
| Poaceae | Poeae | *Festuca* | *Festuca borbonica* | Native | Uncultivated | Perennial |
| Poaceae | Poeae | *Holcus* | *Holcus lanatus* | Introduced | Uncultivated | Perennial |
| Poaceae | Zoysieae | *Sporobolus* | *Sporobolus africanus* | Introduced | Uncultivated | Perennial |

**Supplementary Table 7. Summary of sampled Hemiptera families in each type of site.**

| **Type of Site** | **Site** | ***Aleyrodidae*** | ***Aphididae*** | ***Cicadulina mbila*** | ***Other Cicadellidae*** | ***Delphacidae*** | ***Membracidae*** | ***Miridae*** | ***Psyllidae*** | ***Tropiduchidae*** |
| --- | --- | --- | --- | --- | --- | --- | --- | --- | --- | --- |
| CG | TR | 5 | 6 | 0 | 121 | 269 | 6 | 27 | 4 | 1 |
| AE | BP | 8 | 45 | 101 | 4 | 111 | 11 | 72 | 40 | 11 |
| SG | LE | 0 | 39 | >1000 | 177 | 52 | 1 | 221 | 7 | 0 |
| SG | MV | 0 | 0 | 0 | 4 | 3 | 0 | 35 | 2 | 0 |
| SG | PC | 0 | 3 | 0 | 112 | 9 | 0 | 18 | 1 | 0 |
