# Supplementary figures and images for: Increase of niche filling with increase of host richness for plant-infecting mastreviruses
Source: Virus Evol. 2024 Dec 13;10(1):veae107. doi: 10.1093/ve/veae107 (PMC11665825; doi:10.1093/ve/veae107)

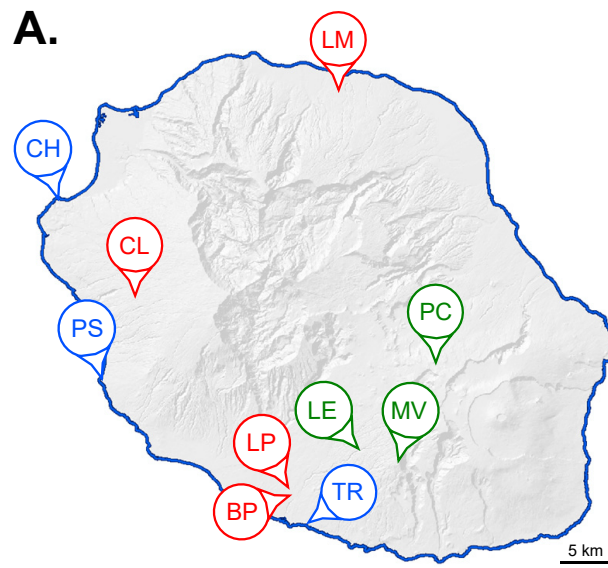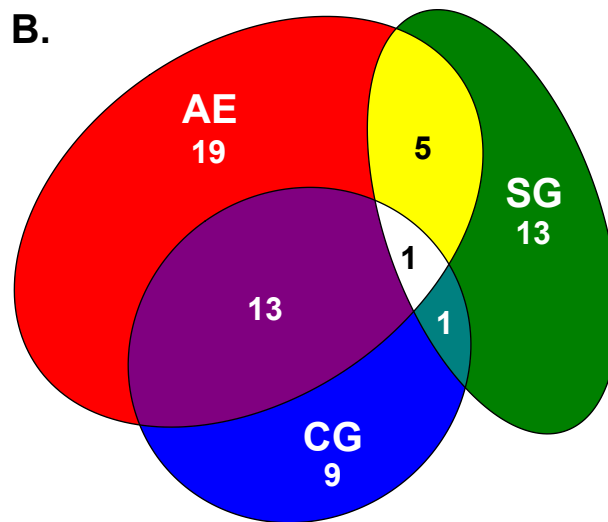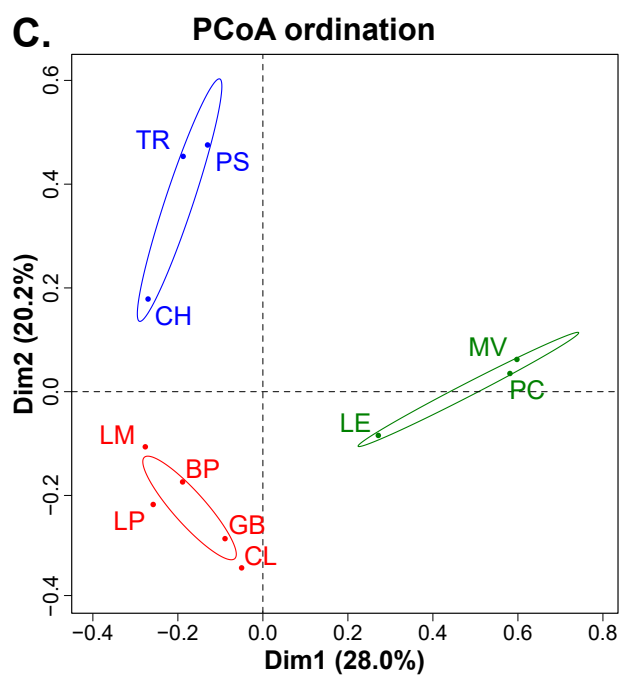

Supplement: veae107_Supp [file veae107_supp.zip › Supp/SUPP_FIGURE_1.pdf]

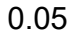

Supplement: veae107_Supp [file veae107_supp.zip › Supp/SUPP_FIGURE_2.pdf]

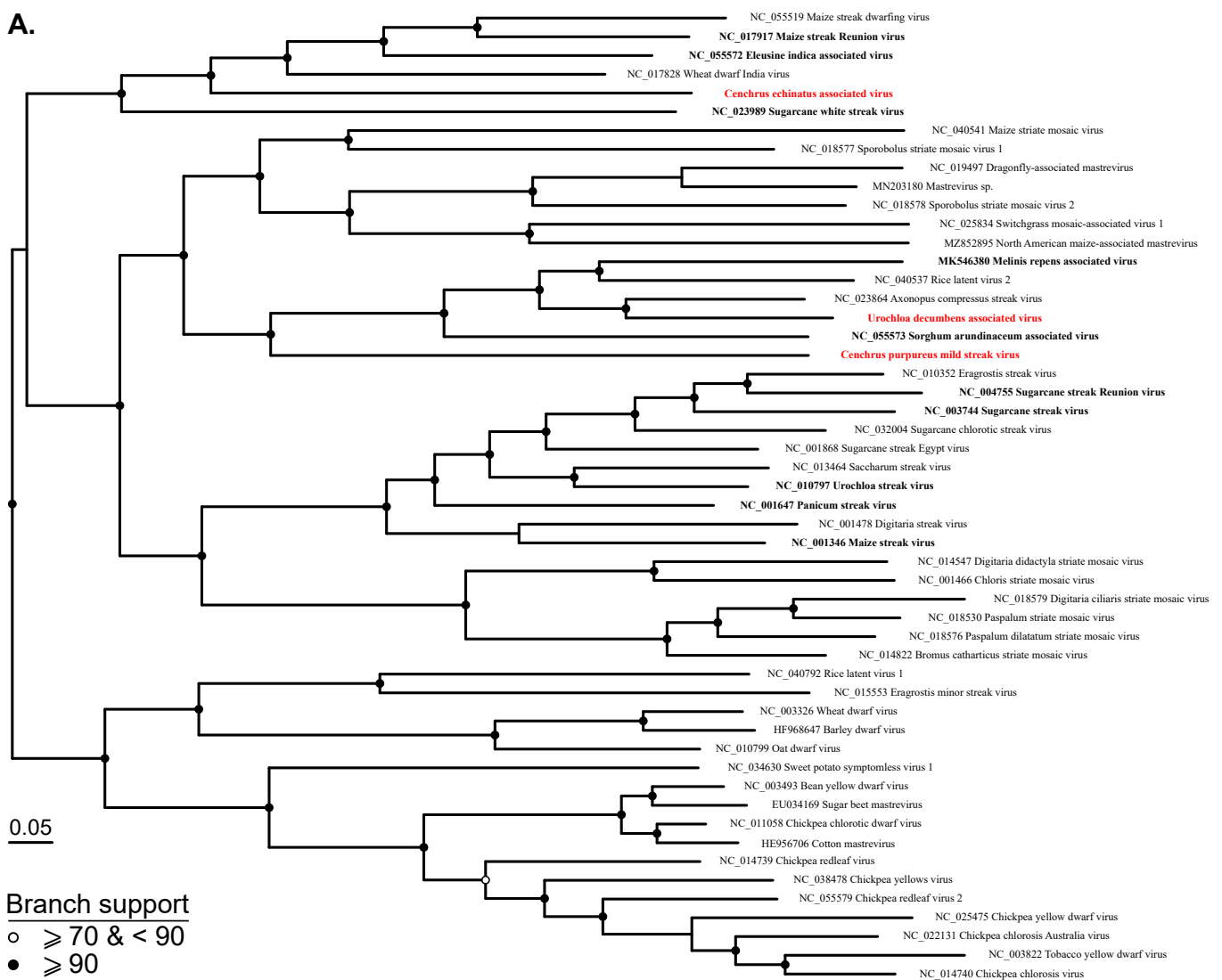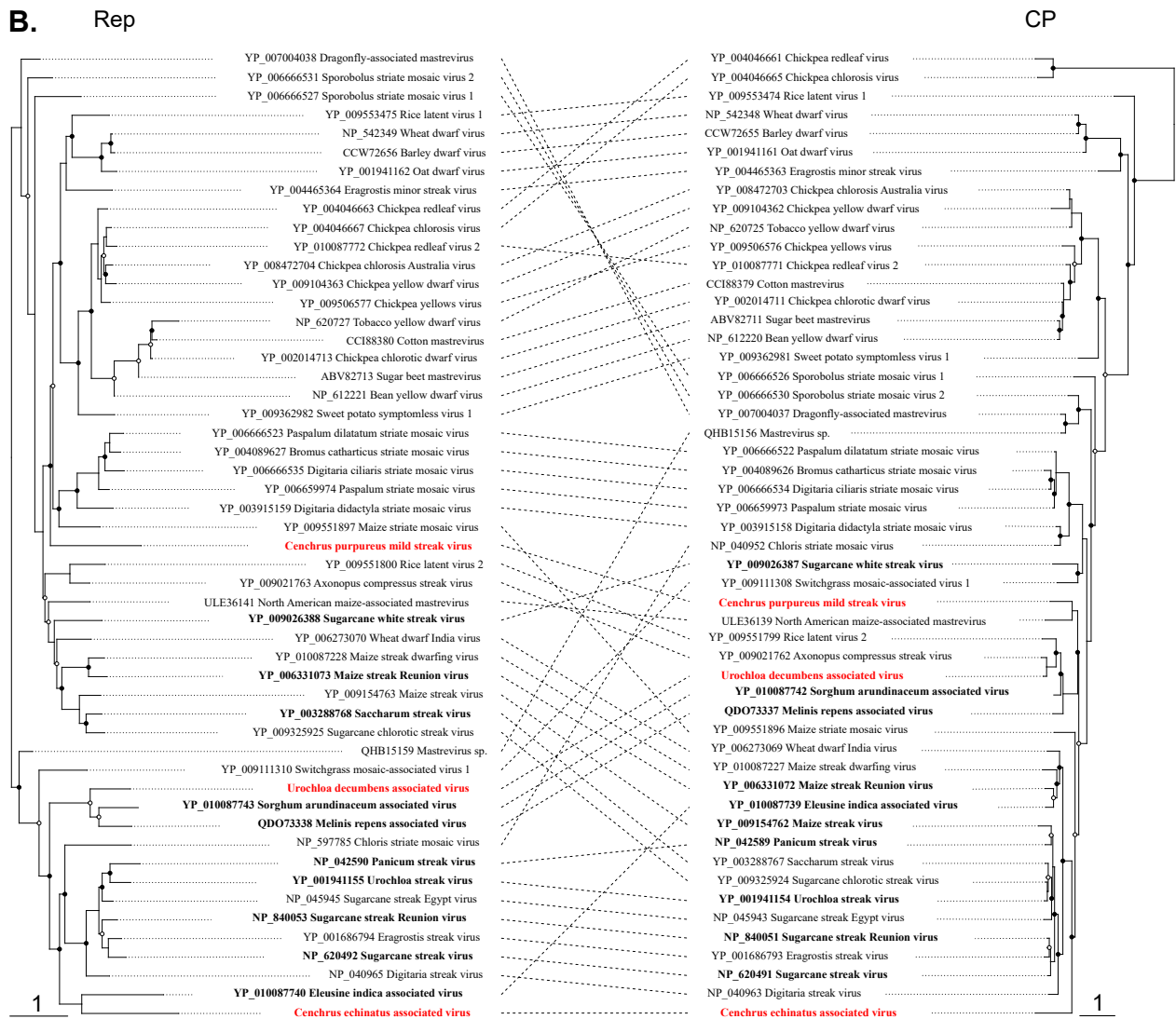

Supplement: veae107_Supp [file veae107_supp.zip › Supp/SUPP_FIGURE_3.pdf]

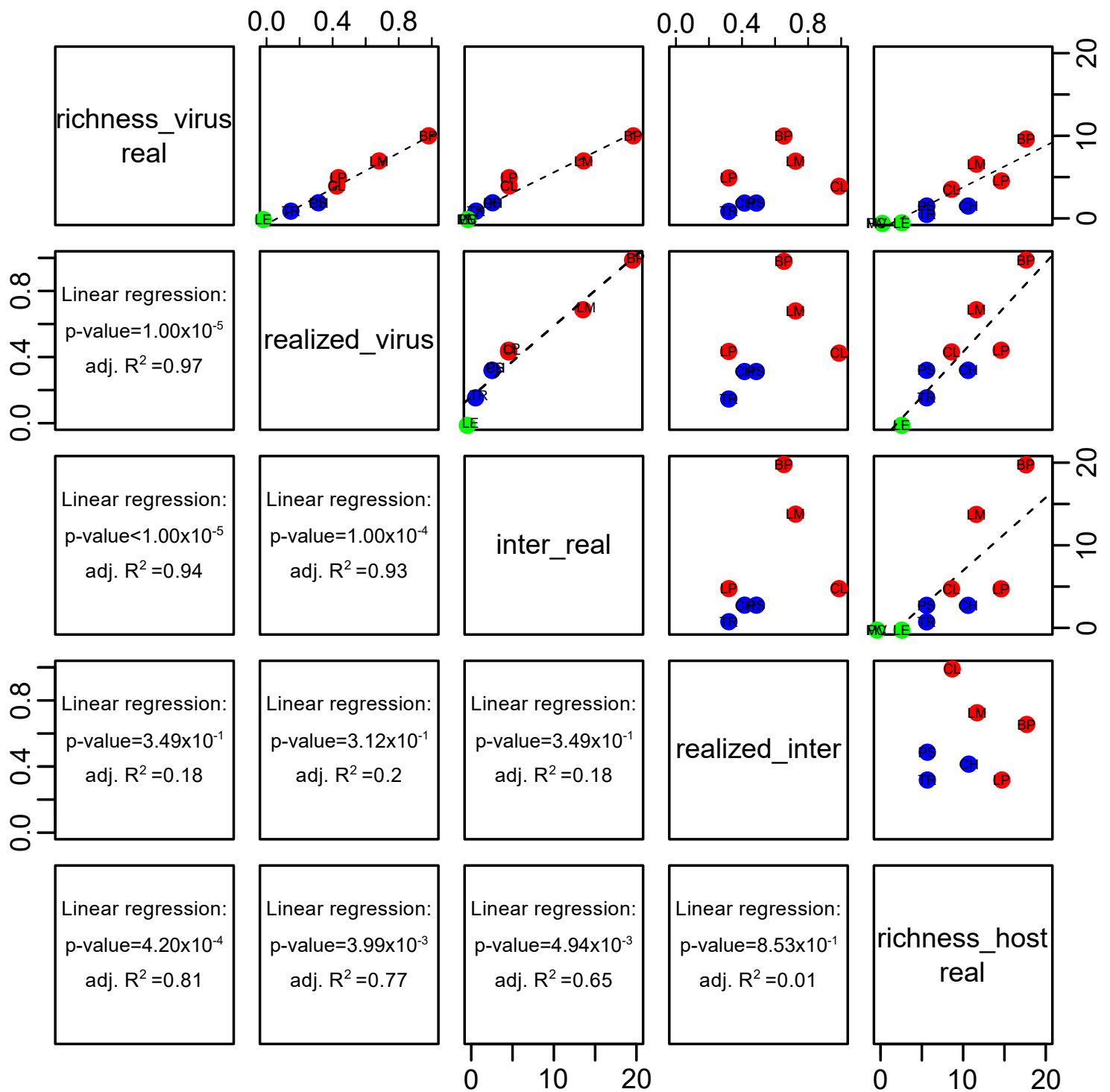

Supplement: veae107_Supp [file veae107_supp.zip › Supp/SUPP_FIGURE_4.pdf]
